# Social learning and memory

**DOI:** 10.1073/pnas.2310033120

**Published:** 2023-08-07

**Authors:** Madeleine Ammar, Laurel Fogarty, Anne Kandler

**Affiliations:** ^a^Theory in Cultural Evolution Lab, Department of Human Behavior, Ecology, and Culture, Max Planck Institute for Evolutionary Anthropology, 04103 Leipzig, Germany

**Keywords:** social learning, memory, cultural variation, adaptation

## Abstract

The adaptability of human populations to changing environments is often attributed to the human capacity for social learning, innovation, and culture. In rapidly changing environments, it has been shown that maintaining high levels of cultural variation is beneficial because it allows for efficient adaptation. However, in many theoretical models, a high level of cultural variation also implies that a large amount of useless and perhaps detrimental information must be maintained and used, leading to lower population fitness in general. Here, we begin to investigate this often conflicting relationship between adaptation and cultural variation. We explicitly allow for the interplay between social learning and environmental variability, alongside the capacity for “memory,” i.e., the storage, retrieval, and forgetting of information. Here, memory allows individuals to retain unexpressed cultural variation, which does not directly impact adaptation. We show that this capacity for memory facilitates the evolution of social learning across a broader range of circumstances than previously thought. Results from this analysis may help to establish whether and when memory should be incorporated into cultural evolutionary models focused on questions of adaptation.

## Significance

One of the most actively debated questions about human and nonhuman culture is this: Under what circumstances might we expect culture, and the ability to learn socially, to be favored by selection? Social learning is thought to be most beneficial when environments change slowly, and innovation when environments change rapidly. We develop a simulation model of social learning incorporating crucial but previously absent aspects of cognition—memory and forgetting. These shape information into useful cultural corpora which, together with high rates of social learning, facilitate rapid adaptation regardless of the rate of environmental change. In summary, we show that the interplay between learning, memory, and forgetting allows the evolution of social learning under conditions where it was previously considered disadvantageous.

The potential of human populations to rapidly adapt to changing environments is often attributed to their capacities for social learning, innovation, and culture (e.g., refs. 1–3). In particular, in frequently changing and recurring environments, it has been shown that high levels of cultural variation allow for efficient adaptation (e.g., ref. 4). However, in most theoretical models, high levels of cultural variation also imply that a, perhaps substantial, fraction of the population carries cultural variants with little or no current benefit. While these variants may become adaptive after an environmental change, they lower the adaptation level of the population in general (5).

Here, we aim to investigate this often conflicting relationship between adaptation and cultural variation. To this end, we develop a simulation model which explores the evolution of social learning in temporally changing and recurring environments when individuals have access to information about cultural variants used in the past. We explicitly allow for the interplay between social learning and innovation, alongside a capacity for “memory,” i.e., the storage, retrieval, and forgetting of information. Here, memory can allow individuals to retain unexpressed cultural variation, which does not directly impact the current level of adaptation. We aim to understand whether such a capacity for memory facilitates the evolution of social learning across a broader range of circumstances than previously thought, and how this feeds back on adaptation. Results from this analysis may help to establish whether and when memory should be incorporated into cultural evolutionary models focused on questions of adaptation.

## The Evolution of Social Learning and Limitations on Cultural Repertoires.

The evolution of social learning has been extensively researched. In most of this work, “social learning” is defined as learning through interaction with conspecifics or their products (6). Social learning is often modeled in opposition to “asocial learning” or innovation, which are typically understood together as any learning that occurs as a result of direct interaction with the environment, in other words, learning in the absence of social interactions with conspecifics. This body of work has resulted in a broad understanding of a range of social learning strategies and mechanisms that render social learning more effective and fruitful (e.g., ref. 7).

One of the most robust and consistent results in this literature suggests that social learning is most useful when the environment is relatively stable and that more innovation is necessary as the environment fluctuates more rapidly (see ref. 8, for a review). This result is intuitive: When the environment changes, social information is likely to be outdated or maladaptive, and the more frequent the changes, the more likely this is to be true. Consequently, a robust inverse relationship exists between the rate of environmental change and the rate of social learning. This relationship, however, relies on certaina ssumptions about the functioning and structure of the individuals’ collection of cultural variants (hereafter, cultural repertoires). Although cultural repertoires are not explicitly referenced in many models of the evolution of social learning, very strong implicit assumptions about their structures are nevertheless present.

For example, in such models, individuals are typically characterized by a single cultural variant. The implicit assumption here is that individuals can recall only one relevant variant: The storage of one completely precludes the storage of any other. Where individuals are assumed to learn such a variant at birth, they typically perform this for the remainder of their lives, and forgetting is impossible. Of course, as with many such assumptions, these are reasonable starting points for the study of social learning. However, it is possible that relaxing these strong assumptions could alter the usually robust interactions between social learning, innovation, and environmental stability which form the basis of our understanding social learning evolution to date.

Some models in this literature do deal with the accumulation of cultural repertoires of different kinds. For example, Fogarty et al. (9) consider the accumulation of selectively neutral cultural variants driven by different social learning mechanisms and population size. However, with its assumption of neutrality, this model was not designed to provide insights into the advantages or disadvantages of cultural repertoires for adaptation. Other kinds of memory have been modeled in the context of neutral cultural evolution. For example, Bentley et al. (10) modeled a kind of population-level memory where individuals could copy cultural information from a number of previous generations. This model addressed the persistence of cultural information through time, but in this context, “memory” did not contribute to the accumulation of individual cultural repertoires but rather allowed the persistence of neutral cultural information for a number of generations. More relevant to our focus on adaptation is the formulation of Costopoulos (11). In this early model, simulated agents could recall the productivity of up to four foraging locations on a landscape. Here, the preliminary analyses produced mixed results but suggested that remembering more locations allowed the agents to adapt to their environments more effectively. Other work has shown that an increase in the size of cultural repertoires, and the ambiguity associated with their payoffs, can lead to a change in the costs and benefits of social learning (12) and affects how individuals explore and exploit their environments (13).

Perhaps some of the most relevant and informative previous work with respect to the model we present below is the “Social Learning Strategies Tournament” (14). This tournament generated a number of insights into the evolution of social learning by allowing simulated agents to accumulate and update information about various cultural variants which could then be strategically deployed. This led to two particularly relevant results. First, more successful strategies relied more heavily on the use of more recent, as opposed to older, information, and second, the ability to use cultural repertoires to evaluate past environmental changes and predict future changes was crucial to success (15). Taken together, these findings hint at an underexplored role for memory in the evolution of social learning.

## Memory and Forgetting.

Learning and memory are different facets of a “unified system” for coding, storing, retrieving, and losing, information about our environment and experiences (16, p. 35). It is intuitive that the acquisition of information is inextricably linked with its storage and retrieval, and it is hard to imagine (or study) one without the other. Likewise, the storage and loss of information over the life of an individual is a complex phenomenon. Indeed, it is widely acknowledged that retaining all available information would not optimize our interaction with the world (for a compelling example, see ref. 17) and so forgetting likely plays a crucial role in such optimization (18). Here, we model social learning, innovation, and forgetting, and to do so, we make a number of simplifying assumptions allowing for a systematic exploration of the interplay between processes. We investigate the accumulation of potentially large repertoires of cultural variants and so assume that individuals can learn and recall a number of relevant pieces of information simultaneously.

We contrast the evolution of social learning in organisms with large cultural repertoires, in which information cannot be lost, with both severely limited repertoires composed of a single variant—most typically modeled in this context, and repertoires shaped by a simple forgetting process.

Modeling forgetting, as with memory itself, is far from straightforward. For our purposes, we define forgetting as a process that can reduce the size of a cultural repertoire. We assume that forgetting rarely used information is more likely on average than forgetting frequently used information, and that information, once forgotten, cannot be retrieved. This simplistic formulation of forgetting warrants some discussion. First, our model is generally agnostic as to the exact cognitive underpinnings of both learning and forgetting, as is typical for models of this kind. This assumption has sparked a lively discussion (see, e.g., ref. 19, for a critical assessment), and the model we present here does little to address these criticisms. However, we stress that our question is evolutionary and the relevant features of forgetting are those that bear directly on our precise question.

We note, too, that distinct forgetting mechanisms have been proposed (see ref. 20). The literature points to two mechanisms in particular, the relative importance and prevalence of which are the subject of some debate. Namely, these are processes that are involved in the decay and loss of information over time (e.g., ref. 20) and those that involve the interference of new information with the storage and retrieval of prior information (e.g., ref. 21). The theory of decay or disuse can be attributed to Thorndike (22) and has been heavily criticized, for example, by McGeoch (23) but has enjoyed a recent revival (e.g., refs. 20 and 24). The, perhaps more widely accepted, theory of interference posits that the acquisition of, and interaction with, new information interferes with the retention of older, similar, information (see, e.g., ref. 21). Our formulation of forgetting is consistent with either process and, under either interpretation, has the evolutionarily relevant effect—the reduction in the size of the cultural repertoires (see below for modeling details).

However, we acknowledge that important differences exist between these processes. The framework we describe below could be straightforwardly tweaked to place more emphasis on, or more accurately model, one process. Here, we opt for a simplistic starting point but acknowledge that further work in developing the model, particularly with regard to its formulation of forgetting, is necessary.

## The Model

### Simulation Framework.

We consider a population of N individuals who experience a temporally changing environment, denoted by E(t). The environment can be in one of two distinct states, i.e., E(t)∈{1,2} for all time steps t, and in each step, the environment can change with probability $p_{\text{env}}$. Each individual aims to express a cultural variant that is well adapted to the currently experienced environment.

Cultural variants are described by the level of benefit, a(k), they convey to their adopters in environmental states k=1,2. It holds 0≤a(k)≤1. As both environmental states are assumed to be distinct, a variant is only beneficial in one of these states, and not the other. Put differently, if a cultural variant provides a meaningful benefit in one environment, it will provide a benefit of close to 0 in the other.

Individuals learn about the level of benefit a cultural variant conveys in environmental state k through social learning or innovation (see below). As we assume that individuals can accurately infer in which environmental state they currently exist, this will generate a precise mapping between environmental state and level of benefit.

Importantly, individuals can maintain knowledge about a number of cultural variants. More precisely, the cultural repertoire of individual j, $M^{j}$, is composed of the benefit values of cultural variants it has socially learned or innovated in its lifetime, including through vertical social learning from parents (see below)

$$M^{j}\left(t\right)=\left[\begin{array}{cccc} a_{1}\left(1\right) & a_{2}\left(1\right) & \ldots & a_{m^{j}}\left(1\right)\\ a_{1}\left(2\right) & a_{2}\left(2\right) & \ldots & a_{m^{j}}\left(2\right) \end{array}\right].$$

The variable $m^{j}$ describes the number of cultural variants present in individual j’s repertoire at time step t. The cultural repertoire is shaped by forgetting (see below), and consequently, information can be lost and the repertoire need not contain all variants an individual has socially learned or innovated throughout over a lifetime.

In addition to its cultural repertoire, an individual is characterized by its genetically inherited propensity, $\xi_{j}$, to engage in social learning, and its genetically inherited propensity, $\varphi_{j}$, to forget knowledge about cultural variants.

#### Expression dynamics.

In each time step, all individuals must express a cultural variant. For that, they go through the following steps.

1. *Choice of variant from cultural repertoire* (hereafter variant choice). An individual j evaluates the benefit levels of all variants present in its repertoire $M^{j}\left(t\right)$ in order to find the variant which may offer the highest benefit in the current environmental state k. More precisely, individual j chooses variant $i\in M^{j}\left(t\right)$ with probability $p_{i}^{j}\left(k\right)$ which is proportional to variant i’s level of benefit $a_{i}\left(k\right)$ relative to the rest of the repertoire in the following way
  [1] $$\begin{array}{c} p_{i}^{j}\left(k\right)=\frac{a_{i}\left(k\right)}{\sum_{s=1}^{m^{j}}a_{s}\left(k\right)}. \end{array}$$
  Decision rule Eq. **1** is motivated by the fact that human decision-making is likely error-prone, and consequently, individuals may not always choose the variant with the highest benefit level from the repertoire for a variety of reasons. However, we note that the field of reinforcement learning which studies optimal decision-making of “goal-directed agents with an uncertain environment” (25, p. 5) has formulated a number of alternative decision rules. In *SI Appendix*, S5, we study the consequences of replacing Eq. **1** with one of these: a softmax rule. We note that many more exist and could be usefully explored in extensions of this model.
2. *Decision regarding learning/innovating.* Individual j then decides whether the anticipated benefit $a_{i}\left(k\right)$ of expressing the chosen variant i is high enough: With probability $a_{i}\left(k\right)$, j expresses variant i and receives a benefit $a_{i}\left(k\right)$; with probability $1-a_{i}\left(k\right)$, it decides to engage in social learning or innovation. In other words, individual j relies on its cultural repertoire—its memory—as long as this repertoire, in combination with decision rule Eq. **1**, provides it with a high level of benefit. Otherwise, individual j seeks novel information through either socially learning or innovating a variant.
  - When *socially learning*, an individual j observes the levels of benefit gained by all individuals during the last time step and chooses to copy a variant proportional to its observed benefit. In other words, it engages in payoff-biased social learning (e.g., ref. 7). Individual j expresses this learned variant, x, receives the benefit $a_{x}\left(k\right)$, and adds x to its repertoire $M^{j}$ (if it is not already contained in it). To explore the impact of this particular social learning process on the cultural dynamic, we replace payoff-biased social learning with unbiased social learning in *SI Appendix*, S4.
  - When innovating, individual j introduces a new variant, y, with a benefit value $a_{y}\left(k\right)$ in the current state k uniformly chosen from the interval between 0 and 1. It expresses this innovated variant, receives benefit $a_{y}\left(k\right)$, and adds y to its repertoire $M^{j}$.
  The balance between social learning and innovation is controlled by a genetically inherited propensity for social learning $\xi_{j}$, i.e., with probability $\xi_{j}$, individual j engages in social learning and with $1-\xi_{j}$ in innovation. Last, we track how often a variant i has been produced by individual j and denote this number of productions by $n_{i}^{j}$.

Following the above steps individual j expresses, in each time step, a particular cultural variant i and receives the benefit associated with this variant in environmental state k, $a_{i}\left(k\right)$. In contrast to many reinforcement learning problems, expression is assumed to be error-free.

#### Forgetting.

In each time step, it is also possible for individuals to lose knowledge. With its genetically inherited propensity for forgetting $\varphi_{j}$, individual j removes one cultural variant from its repertoire $M^{j}$ with the restriction that the variant expressed in the current time step cannot be forgotten. This restriction precludes the possibility that individuals could have an empty repertoire. More precisely, if individual j “decides” to forget, it “chooses” variant z to be removed from its repertoire with a probability, $f_{z}^{j}$, that is inversely proportional to the number of times j has expressed z so far, $n_{z}^{j}$. This probability is given by

[2] $$\begin{array}{c} f_{z}^{j}=\frac{A-n_{z}^{j}}{\sum_{\begin{array}{c} s=1\\ s\neq i \end{array}}^{m^{j}}\left(A-n_{s}^{j}\right)}, \end{array}$$

where $A=\sum_{\begin{array}{c} s=1\\ s\neq i \end{array}}^{m^{j}}n_{s}^{j}$ and i denotes the variant expressed in this time step.

Summarizing, the probability $\varphi_{j}$ determines how frequently individual j forgets one variant from its repertoire: Low values of $\varphi_{j}$ maintain most of the socially learned or innovated cultural variants in j’s repertoire, while high values of $\varphi_{j}$ lead to frequent forgetting of rarely expressed variants. We stress that Eq. **2** is not the only plausible choice for a forgetting rule. In *SI Appendix*, S3, we study the consequences of replacing Eq. **2** with “random” forgetting, i.e., when variants to be forgotten are randomly chosen from the repertoire, and “bad variant” forgetting whereby variants with low benefits are predominantly forgotten.

#### Birth–death process and vertical social learning.

Finally, in each time step, one individual is chosen for reproduction and one for death. While the reproducing individual is chosen proportionally to the benefit accrued in this time step, death is age-dependent. The probability that an individual dies is proportional to its relative age such that older individuals are more likely to die than younger individuals. This allows us to more clearly isolate the effects of vertical social learning. If individual j is chosen to reproduce, it passes its ξ value and φ value to its offspring. This happens faithfully with a probability $1-\mu_{\xi }$ and $1-\mu_{\varphi }$, respectively. With probability $\mu_{\xi }$, the offspring will possess

$$\xi_{j}+\varepsilon \;\text{with}\;\varepsilon ∼N\left(0,\sigma_{\xi }^{2}\right),$$

and with probability $\mu_{\varphi }$

$$\phi_{j}+\varepsilon \;\text{with}\;\varepsilon ∼N\left(0,\sigma_{\varphi }^{2}\right).$$

Note that the normally distributed errors are truncated to avoid values larger than 1 or smaller than 0.

To allow for intergenerational transmission of knowledge, we assume that individual j passes on its full repertoire $M^{j}$ to the naive individual. This means that the offspring inherits the benefit level of all variants in its parent’s repertoire, but it does not inherit information about the parent’s experiences of the traits in the repertoire—the $n_{i}^{j}$ values describing the number of times a variant was expressed by the parent. Thus, at first, all vertically learned variants are equally susceptible to forgetting as their numbers of expressions, $n_{i}^{j}$ are set to 1. We note that this is an assumption that acts against the benefits of memory in the sense that a newborn individual could, with a certain probability, forget a number of highly beneficial variants in their first time steps.

#### Simulation setup.

Simulations are composed of single populations consisting of N=200 individuals and were run for 100,000 time steps each (i.e., for 500 generations) which ensured that an equilibrium has been reached. Initially, all individuals have a propensity for social learning of 0.2 and a propensity for forgetting of 0.2. Further, to initialize the simulation, each individual present at time t=0 innovates a variant and expresses it. In the analyses shown below, unless otherwise stated, we set $\mu_{\xi }=0.05$, $\sigma_{\xi }^{2}=0.1$, $\mu_{\varphi }=0.05$, and $\sigma_{\varphi }^{2}=0.1$ and ran 300 simulations for each level of environmental instability $p_{\text{env}}\in \left\{0,0.0001,0.001,0.005,0.0075,0.01,0.05,0.1,0.2\right\}$.

## Results

To understand the interplay between social learning, memory, and environmental instability, we use the simulation model described above to analyze three distinct scenarios involving populations of individuals with

(i) limited repertoires consisting of a single cultural variant only,
(ii) repertoires consisting of all cultural variants individuals have socially learned or innovated in their lifetime, and no possibility of forgetting,
(iii) repertoires shaped by the forgetting processes.

**Fig. 1.**
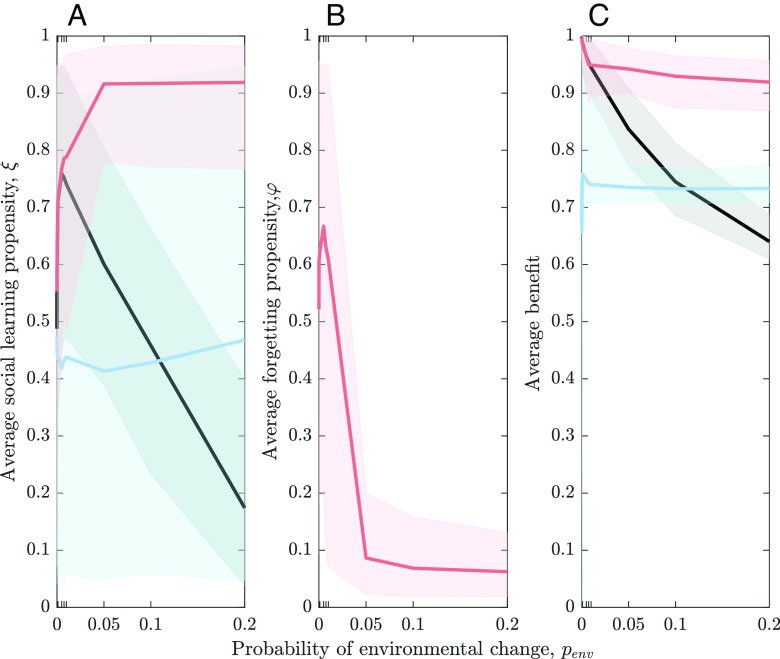
Relationship between rates of environmental change $p_{\text{env}}$ and (*A*) propensity for social learning, (*B*) propensity for forgetting, and (*C*) population-level benefit averaged over all individuals in the last time step for 300 independent simulations. Shaded areas represent 95% CIs of population averages. Scenario (i) is shown by black lines, scenario (ii) by blue lines, and scenario (iii) by light pink lines.

Scenario (i) largely reflects the situation considered by most existing models on the evolution of social learning in temporally unstable environments (e.g., refs. 26–31). However, our framework allows additionally for vertical social learning: offspring inherit their parents’ cultural variant. Nevertheless, we recover the expected result: the more temporally unstable the environment (as quantified by increasing values of $p_{\text{env}}$), the lower the evolved rate of social learning (as quantified by the population-level propensity to engage in social learning, $\overset{¯}{\xi }$) (see black lines in Fig. 1*A*).

Scenario (ii) allows for the accumulation of all socially learned and innovated cultural variants. For computational reasons, we restrict the size of the individual repertoires to 500. Once this limit is reached, no further social learning or innovation can occur and so no variant can be added. Fig. 1*A* (blue line) shows that the maintenance of large cultural repertoires disrupts the relationship between environmental instability and the propensity for social learning: The population-level propensity for social learning, $\overset{¯}{\xi }$, is now largely independent of environmental instability.

Finally, scenario (iii) allows forgetting to adjust the size of individual cultural repertoires. Fig. 1*A* shows that this effectively reverses the relationship between social learning and environmental instability: The average population-level propensity for social learning, $\overset{¯}{\xi }$, now increases with environmental instability and then stays at a high level (see the pink line in Fig. 1*A*).

In the sections that follow, we explore the model mechanisms underpinning these results.

### Scenario (i): Adaptation to a Fluctuating Environment with Limited Memory.

**Fig. 2.**
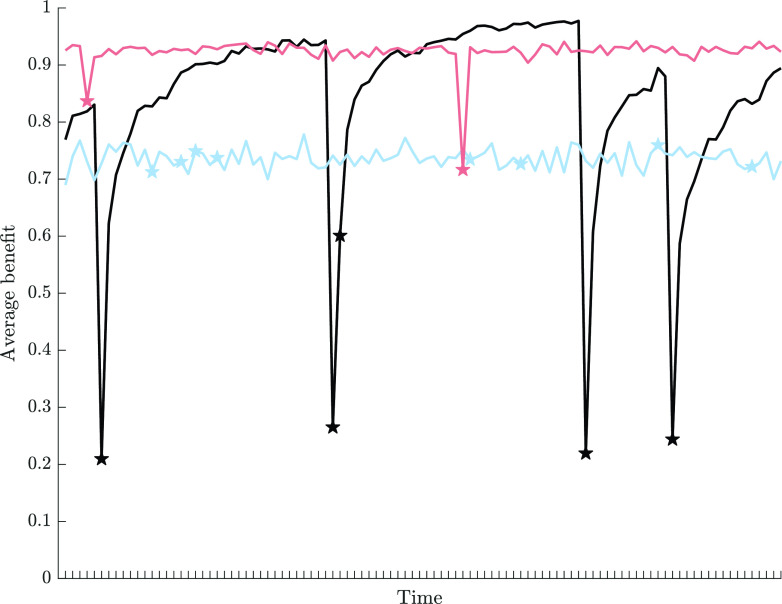
Average benefit received through variant expression over all individuals (N=200) in a single simulation at each time step. Results are shown for scenario (i) (black line), scenario (ii) (blue line), and scenario (iii) (light pink line) at an environmental instability $p_{\text{env}}=0.05$. In each case, time steps in which environmental changes occurred are indicated by stars of corresponding colors.

In populations where individuals can only possess a single cultural variant at any one time, social learning has some obvious disadvantages. These are especially clear in the time steps directly following an environmental change. Fig. 2 (black line) shows the temporal change in the population benefit level during a single representative simulation run for $p_{\text{env}}=0.05$, i.e., for on average 10 environmental changes in an individual’s average lifetime. While the benefit level is high in stable periods, it drops severely at every environmental change. This happens because individuals now acquire mostly outdated information through social learning. Individuals must generate cultural variants adapted to the new environmental state through innovation which then can spread via social learning through the population (32).

This dynamic drives the relationship between social learning and environmental instability: The more unstable the environment, i.e., the higher $p_{\text{env}}$, the more beneficial innovation becomes, and, consequently, the lower the propensity for social learning, which might spread outdated information (see Fig. 1*A*, black line).

This affects the average benefit level of the population shown in Fig. 1*C* (black line). When environmental changes are frequent, there are more frequent drops in the benefit level and a shorter time available for recovery. This leads to lower benefit levels in the (shorter) stable periods and consequently lower average levels of benefit for the population. For $p_{\text{env}}=0$, one might expect that social learning propensity, $\overset{¯}{\xi }$, would be high or even 1. In our model, however, the relatively low value of $\overset{¯}{\xi }$ for $p_{\text{env}}=0$ in Fig. 1*A* is caused by vertical social learning: When offspring experience the same environmental state as their parents, the inherited cultural variants remain beneficial, and individuals rarely engage in social learning or innovation. The propensity of social learning evolves in this case mainly through drift.

### Scenario (ii): Adaptation to a Fluctuating Environment without Forgetting.

In populations where individuals can remember all socially learned or innovated cultural variants (at least until the limit of 500 is reached), the benefit level of the population stays roughly constant over time, regardless of how frequently the environment is changing (see Fig. 2, blue line for $p_{\text{env}}=0.05$). There is no drop in the population benefit level at an environmental change. However, during environmentally stable periods, benefit levels are, in general, lower than in scenario (i) resulting in lower average benefit levels (see Fig. 1*C*, blue line). Further, the population-level propensity for social learning is only weakly influenced by environmental instability. This is partly a result of the limit imposed on the repertoire sizes: Once the limit is reached, no further learning or innovation events are possible, meaning that $\overset{¯}{\xi }$ evolves largely through drift. However, we note that any reasonable limit on the repertoire size would have a similar effect.

**Fig. 3.**
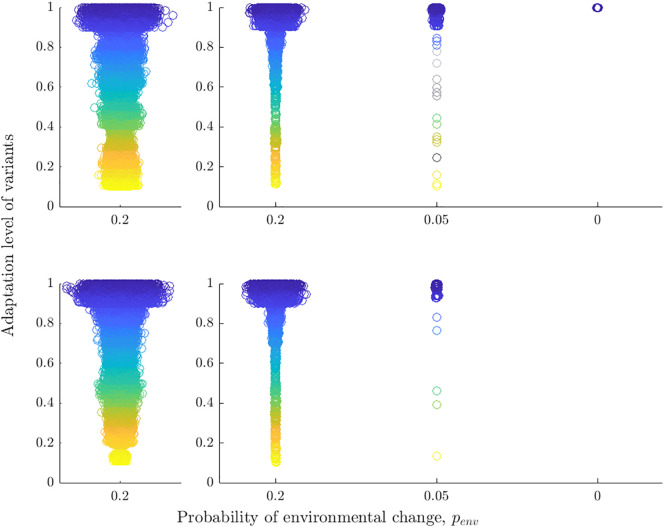
Cultural repertoires for different levels of environmental instability $p_{\text{env}}$. Open circles represent variants of a particular adaptive value (low adaptive values are shown in yellow, and high adaptive values are shown in blue). Results are shown for all individuals (N=200) in the last time step of a single simulation, and variants are categorized as being (*Top row*) adapted to the current environmental state or (*Bottom row*) adapted to the environmental state the population was not exposed to in the last time step. Dots are jittered to avoid overlapping points. Thus, the breadth of the “cloud” indicates the presence of many variants of a particular adaptive value. The first column describes the repertoires for scenario (ii), and the second column for scenario (iii).

To understand the relationship between environmental instability and level of benefit shown in Figs. 1*C* and 2, we examine the structure of the repertoires. When individuals cannot forget, all repertoires provide high benefits in both environmental states. However, they also possess a sizable number of variants that provide low or medium benefits (see Fig. 3, first column, but we note that lower $p_{\text{env}}$ values lead to similar structures). Thus, when choosing a variant for expression from their repertoires according to Eq. **1**, individuals will almost always choose a “good” cultural variant but rarely the “best” one—the one with the highest adaptation level contained in their repertoire.

**Fig. 4.**
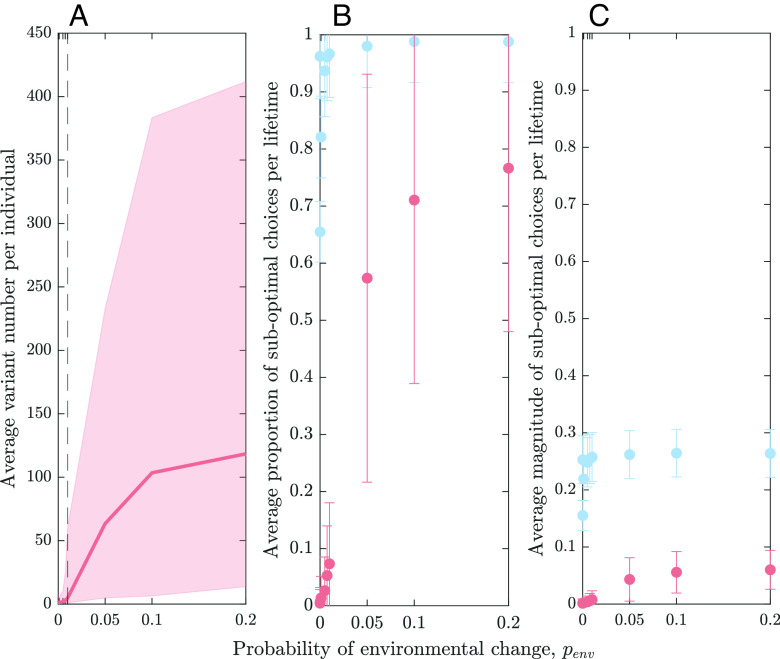
Relationship between rates of environmental change $p_{\text{env}}$ and (*A*) the size of individual repertoires, (*B*) the proportion of suboptimal choices, and (*C*) the magnitude of suboptimal choices relative to the most adaptive variant available in an individual’s repertoire. Lines and dots represent the average across all individuals (N=200) living in the last generation over 300 independent simulations. Shaded areas represent 95% CIs of population averages. Error bars indicate the SD of population averages. The dashed vertical line in panel (*A*) marks the boundary between the “stable” and “unstable” range discussed in the text. Results are shown for scenario (ii) (blue) and scenario (iii) (light pink). In panel (*A*), values for scenario (ii) are always 500, and therefore, the line is not shown.

To illustrate this point, we analyze the expression choices of all individuals in the population for different $p_{\text{env}}$ values. Fig. 4*B* (blue filled circles) shows that the fraction of choices of the population that led to “suboptimal” outcomes, i.e., the choice of a variant other than the “best” one, is very high for all rates of environmental change. Additionally, Fig. 4*C* (blue filled circles) shows that the average difference between the chosen and “best” variant in the repertoire is also high, again, for all rates of environmental change. This explains the low levels of benefit shown in Figs. 1*C* and 2 (blue lines) and also, partly, the independence of the levels of benefit from the rate of environmental change: It simply does not matter how frequently the environment changes if individuals have variants providing benefits in both environmental states in their repertoires.

We note that this dynamic is, in part, driven by decision rule Eq. **1**. If individuals are able to choose highly beneficial variants more reliably, then the number of “suboptimal” choices and the magnitude between the chosen and “best” variant in the repertoire become smaller. This, in turn, leads to higher average benefit levels in the population. In other words, it is the combination of the structure of the repertoire and the decision rule used for choosing a variant from this cultural repertoire which determines the average population benefit level.

Summarizing, assuming that each environmental state has been experienced during an individual’s lifetime as they build their repertoires, large repertoires constructed by remembering every socially learned or innovated cultural variant shield individuals from drops in the benefit level at environmental changes occurring at any frequency. However, this comes at the cost of a lower average benefit level for the population if individuals employ error-prone decision rules, as, e.g., given by Eq. **1**, for choosing a variant from their repertoire.

### Scenario (iii): Adaptation to a Fluctuating Environment with Memory Including Evolving Rates of Forgetting.

In populations where individuals can construct their cultural repertoires through a combination of social learning and innovation, and forgetting, benefit levels are consistently high with relatively small drops after an environmental change (see Fig. 2, light pink line, for $p_{\text{env}}\;=\;0.05$). This is true, again, for any rate of environmental change, and we see high average levels of benefit that are not greatly influenced by $p_{\text{env}}$ (see Fig. 1*C*, light pink line). This dynamic is driven by a finely tuned interplay between social learning and forgetting: The propensity for social learning increases to a level close to 1 when the environment is unstable (see Fig. 1*A*, light pink line), while the propensity for forgetting first increases and then, when environmental changes are likely to occur multiple times in an individual’s lifetime, decrease to a low level close to, but not exactly, 0 (Fig. 1*B*).

To understand these patterns, we examine the structure of the repertoires in two environmental change ranges: one in which environmental changes are likely to occur multiple times in an individual’s lifetime (the “unstable range” with values of $p_{\text{env}}$ larger than 0.01) and one where environmental changes occur less often (the “stable range” with values of $p_{\text{env}}$ smaller than or equal to 0.01).

When $p_{\text{env}}=0$, almost all repertoires consist of a single, nearly perfectly adapted variant leading to an average benefit level in the population of almost 1. Under these conditions, social learning and innovations are very rare. Thus, the propensities for social learning and forgetting evolve through drift leading to average values in the population of around 0.5.

If the population experiences occasional environmental changes, high propensities for forgetting still restrict the average size of an individual’s repertoire (Fig. 4*A*), but the number of social learning or innovation events increases. This leads to an increase in average social learning propensity, and this increase is slightly higher in scenario (iii) than in scenario (i) (cf. light pink and black lines in Fig. 1*A*, but note the overlapping CIs). At the same time, the propensity for forgetting stays above 0.5. As in scenario (i), social learning is preferred over innovation, and the relatively high rate of forgetting ensures that the average size of an individual’s repertoire stays small. Nevertheless, there are some individuals with more than one cultural variant generating unexpressed population-level cultural variation. Importantly, for $p_{\text{env}}>0.0001$, this unexpressed variation may also contain variants adapted to the alternative environmental state directly prior to an environmental change (Table 1). This reduces the need for innovation after an environmental change as social learning can act on the existing standing variation. In other words, the population does not need to invent novel adaptive cultural variants: These can be recalled from the individual repertoires containing them, and the high propensity for social learning in the populations leads to their rapid spread.

**Table 1.** Relationship between environmental instability ($p_{\mathrm{env}}$), the average number of individuals exhibiting a variant that is adapted to the alternative state directly prior to an environmental change ($N_{\mathrm{adapt}}$), and the average adaptation level of those variants ($V_{\mathrm{adapt}}$)

$p_{\text{env}}$	$N_{\text{adapt}}$	$V_{\;}\text{adapt}$
0.0001	0	0
0.001	0.45	0.6512
0.005	3.20	0.8738
0.0075	5.24	0.8827
0.01	6.16	0.8484
0.05	127.51	0.8916
0.1	163.66	0.8717
0.2	181.71	0.8652

In the unstable regime, we observe a very different dynamic. While the propensity for social learning further increases with $p_{\text{env}}$, the propensity for forgetting decreases leading to a substantial increase in the sizes of individual repertoires. For $p_{\text{env}}=0.05$, the average repertoire is composed of around 60 variants, but importantly, this means that the majority of individuals now possess at least one variant highly adapted to the other, not currently experienced environmental state directly prior to an environmental change (Table 1). Furthermore, these repertoires are highly structured: They contain almost exclusively highly adaptive variants adapted to both environmental states generating unexpressed but nonetheless beneficial cultural variation (Fig. 3). This reduces the need for both social learning and innovation, especially at the point of an environmental change. If individuals decide to engage in learning or innovation, they typically learn socially as the probability of obtaining a highly adaptive variant is considerably higher via social learning than via innovating. Compared to the stable regime, we observe only a small decrease in the adaptation level of the population.

But why should the individual repertoires evolve to contain, on average, more than two cultural variants—one for each environmental state? These larger repertoires buffer against the random loss of highly adaptive variants in either environmental state through the forgetting process. Even though the increased size leads to a higher fraction of suboptimal choices from the repertoire (as discussed above), there is a balance—highly structured repertoires ensure, in contrast to scenario (ii), that the consequences of the suboptimal choices for the overall adaptation level are small (see Fig. 4 *B* and *C*, light pink circles).

In summary, the interplay between choosing a variant from individual repertoires according to Eq. **1**, social learning, innovation, and forgetting generates highly structured individual cultural repertoires. These repertoires are characterized by a number of cultural variants which can be adapted to either environmental state, and their structure depends on the rate of environmental change. In particular, the forgetting processes erase rarely used (and often poorly adapted) variants, producing repertoires that are biased toward highly beneficial variants. This means that the population has access to substantial cultural variation which, importantly, is rarely expressed and therefore does not have a detrimental effect on the current benefit level of the population. This largely unexpressed variation provides the basis for social learning to be useful for any rate of environmental change: At a change, individuals can express highly beneficial variants from their cultural repertoires. This mitigates the risk of observing or learning outdated information.

The highly structured repertoires can mitigate the errors caused by using decision rule Eq. **1** for choosing a cultural variant from the repertoire: If the repertoires consist of mainly highly adaptive variants, the magnitude of suboptimal choices is greatly reduced (see Fig. 4). If, however, the decision process is less error-prone as, e.g., captured by a softmax rule with low temperature (*SI Appendix*, S5), there is no need for structured repertoires as the decision rule itself leads individuals to consistently choose highly adaptive variants. Indeed, we see that replacing decision rule Eq. **1** with a softmax rule with low temperature leads to slightly lower forgetting propensities than shown in Fig. 1*B*. This in turn results in larger individual repertoires that are less structured, i.e., they contain more poorly adapted cultural variants. Nevertheless, the more accurate decision rule leads to an even smaller magnitude of suboptimal choices (*SI Appendix*, Fig. S15) and consequently higher average benefit levels of the population (*SI Appendix*, Fig. S13).

Finally, we turn our attention to the role of vertical transmission or vertical social learning. For low $p_{\text{env}}$values, environmental changes may not happen within an individual’s lifetime, and whole generations might live and die in the same environmental state. In our model, vertical social learning is the only process capable of maintaining cultural variants adaptive to the alternative environmental state for some considerable time after an environmental change. Without vertical social learning, all information about the other state would be lost, at the latest, with the death of the last individual that had experienced the other state.

Table 1 shows the fraction of the population that possesses a variant adaptive to the alternative state directly prior to an environmental change. This number starkly decreases when the environment is more stable pointing to the limits of vertical social learning, and our formulation of it in particular, in maintaining diverse knowledge relevant to either environmental state. Nevertheless, even if only one individual carries knowledge about the alternative state, this forms, together with the high propensity for social learning, a population-level “cultural repertoire” that allows for rapid adaptation.

## Discussion

One of the most consistent and fundamental results in the evolution of social learning literature is the inverse relationship between the rate of environmental change and the benefit of social learning, as opposed to innovation (e.g., refs. 8, 26, and 33). This result has been derived under the assumption that individuals can store just one cultural variant at any time and, consequently, learning or innovating a new variant implies forgetting the “old” one. In this case, environmental change necessitates innovation: social learning is insufficient because all, or most, individuals will possess only outdated information. The model presented in this paper allows for the generation of cultural repertoires, i.e., an individual collection of cultural variants, through simple formulations of learning, innovation, and forgetting processes. The model, therefore, generalizes previously restrictive assumptions about memory and allows us to begin to unpack the complex role of memory and individual decision-making in the evolution of social learning.

Under the assumption that individual decision-making is error-prone, i.e., individuals frequently choose suboptimal variants from their cultural repertoires, we found that those repertoires, once shaped by forgetting and social learning, can act in concert with high rates of social learning to maintain high benefit levels in the population, even in unstable environments. In other words, if individuals are equipped with the capacity to accumulate and use cultural repertoires, the usually robust relationship between the rate of environmental change and the benefit of social learning is altered: High rate of social learning is adaptive regardless of the rate of environmental change. This result is driven by the ability of the agents to accumulate and maintain cultural variation that need not be expressed, including cultural variants adapted to environmental states different to the current one.

However, this does not mean that individuals should remember all cultural variants. In the case where forgetting is impossible, individuals are likely to accumulate, in a very short amount of time (in the first 4% of simulation runtime), a very large number of cultural variants appropriate for either environment. This shields individuals from drops in the adaptation level at environmental changes occurring at any frequency. However, this comes at the cost of a lower average benefit level for the population. Just as individuals cannot forget good variants, they cannot forget bad ones either. Although, in our model, individuals are likely to choose these bad variants to express with a lower probability, choosing them is still a possibility. Where there are many such possibilities, the probability of choosing the “best” available variant is low, and the average adaptation level of the population is reduced as a result.

These results suggest a vital and positive role for forgetting. With simple heuristics for forgetting, our model suggests that the interplay between social learning, innovation, and forgetting generates highly structured individual cultural repertoires, leading to consistently high benefit levels. Repertoires contain a set of cultural variants, and the count of variants adapted to either environmental state depends on the rate of change. Thus, the population has access to substantial unexpressed cultural variation which does not have a detrimental effect on the current benefit level of the population. Unexpressed variation allows social learning to be useful at any rate of environmental change: At a change, individuals can express beneficial variants from their repertoires. This mitigates the risk of learning outdated information. While the rate of social learning remained high in all environmental change regimes, the rate of forgetting interacted with the level of environmental stability to generate individual repertoires containing a sufficient level of cultural variation.

These results also suggest that the structured repertoires have evolved to reduce the detrimental effect of error-prone decision rule Eq. **1**. If individuals choose from a repertoire of mainly highly beneficial cultural variants, then the precise decision rule becomes less important. However, under the assumption that individual decision-making is less error-prone, highly structured repertoires do not evolve as individuals are able to reliably identify the highly beneficial variants in their repertoire regardless of its composition. This is not to say that forgetting is not needed in this situation—forgetting propensities evolve to only slightly lower levels as under error-prone decision-making leading to slightly higher average repertoire sizes.

Interestingly, we observe that for lower rates of environmental change, the average size of individual repertoires is small. Nevertheless, directly prior to an environmental change, it is still likely that a small number of individuals carry variants adapted to the other environment. This “population-level memory” in concert with the high propensity for social learning leads to extremely rapid adaptation (Fig. 2), even where most individual repertoires is small. However, for higher rates of environmental change, the average size of the individual repertoires increases, and the majority of individuals possess at least one variant highly adapted to the other, not currently experienced environmental state at an environmental change. Individuals now frequently rely on their own “individual-level memory” resulting in similarly rapid adaptation (Fig. 2). This mirrors previous results that cultural adaptation from standing variation is often considerably faster than adaptation from innovation alone (5).

We note that neither changes in the process of social learning from payoff-biased to unbiased nor changes in the forgetting process from Eq. **2** to “random” or “bad variant” forgetting changed the dynamics of the system qualitatively.

Last, we stress that we focused on periodically changing environments, and our results hold only in cases where populations experience identical or similar environments repeatedly. More precisely, we distinguish between two environmental states which may correspond to coarse categories like “hot” and “cold” or “wet” and “dry”. Given this interpretation, our assumption that individuals can correctly infer the environmental state they currently exist in is justifiable. However, future work should explore the consequences of both, error-prone inference of the environmental state and the inclusion of more than two environmental states.

Further, our results have been derived under the assumption that innovations are very likely to produce cultural variants with some positive adaptation level—as described above, the adaptation level of any innovated cultural variant is randomly chosen from the interval 0 and 1. In other words, innovations are likely to provide at least some benefits. We also severely restricted the potential long-term effects of vertical social learning by assuming that all vertically transmitted variants are equally susceptible to forgetting. In short, we evaluated the interplay between social learning, innovation, and memory processes under assumptions that disadvantage social learning. Nevertheless, social learning, in combination with finely tuned cultural repertoires, evolves to high rates in all environmental change regimes that we considered. The consequences of relaxing these assumptions should be explored in future work.

Summarizing, relatively high benefit levels can be maintained in the absence of memory through the strategic use of social learning and innovation. But even higher levels can be maintained through consistently high rates of social learning and the strategic use of memory and forgetting, especially in unstable environments. Forgetting as implemented here not only improves the benefits of social learning but also has the potential to shape large and costly accumulations of information into useful, adaptive, and unexpressed cultural repertoires. Our results point to the necessity of including relevant cognitive processes such as memory in models in order to understand the evolution and maintenance of social learning and, ultimately, the benefits of culture.

## Supplementary Material

Appendix 01 (PDF)

## Data Availability

Code data have been deposited in SocialLearning Memory (34).
