# Supplementary material for: Social learning and memory
Source: Proc Natl Acad Sci U S A. 2023 Aug 7;120(33):e2310033120. doi: 10.1073/pnas.2310033120 (PMC10433305; doi:10.1073/pnas.2310033120)
Supplement: Supplementary file 1 — Appendix 01 (PDF) [file pnas.2310033120.sapp.pdf]

Supplementary Material to Social Learning and Memory

Madeleine Ammar[1], Laurel Fogarty[1], and Anne Kandler[1]

[1]TICE lab, Department of Human Behavior, Ecology, and Culture, Max Planck Institute for Evolutionary Anthropology, Deutscher Platz 6, 04103 Leipzig

July 13, 2023

Corresponding Author: Madeleine Ammar

Email: madeleine.ammar@eva.mpg.de

This PDF includes:

- Supporting text
- Figures S1 to S19

S1 Individual Development

The process of vertical social learning described in Section 2.1.3. in the main text leads to the transmission of the full cultural repertoire from parent to offspring. In other words, an offspring inherits the benefit level of all variants in its parent's repertoire and therefore does not need to build up its own repertoire at the beginning. However, it does not inherit the n_i^j values — the number of times a variant was expressed by the parent. All vertically transmitted cultural variants are assumed to be expressed once at the beginning of an individual's life. Processes of social learning/innovation and forgetting will change the composition of the individual repertoires over an individual's life time. Nevertheless, the general structure, i.e. the ratio between highly and poorly adapted cultural variants does not change substantially. What of course is changing over the life time is number of times a variant was expressed. Fig. S1 illustrates this for two exemplary individuals: they show how the number of expressions of variants with different adaptive values change over the life time of those individuals for $p_{\text{env}} = 0.05$. We see that highly adaptive variants are expressed more frequently but also that large repertoires in combination with decision rule (1), given in the main text, can lead to expressions of variants with a low adaptive values.

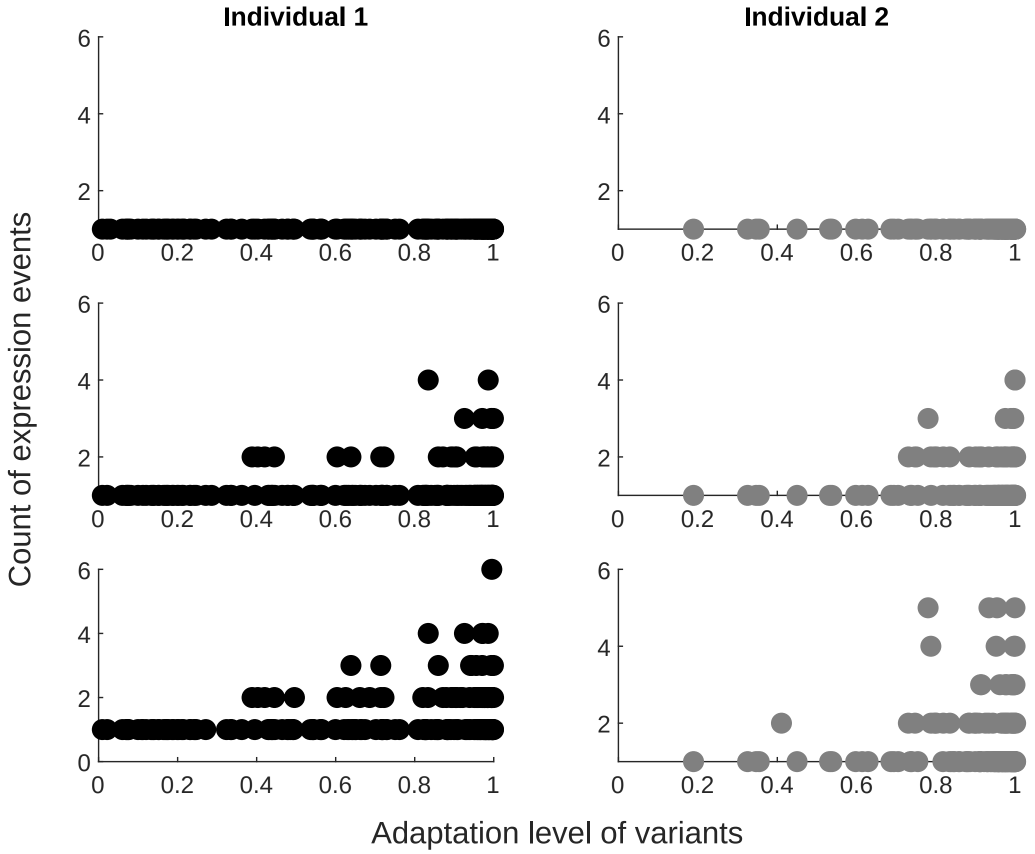

Figure S1: Cultural repertoires of two individuals in different life stages (top panels: age 1, middle panels: age 50, bottom panels: age 100) for $p_{\text{env}} = 0.05$. Dots indicate the count of expression events for variants of a given adaptation level.

S2 Drift and Population Size

In the main text we contrasted the evolution of the average propensity for social learning in populations where individuals shape their cultural repertoires through forgetting (scenario iii.) with the evolution in populations where individuals maintain all expressed variants in their cultural repertoire (scenario ii.). We argued that in scenario ii. the propensity for social learning evolves largely through drift. To corroborate this claim Fig. S2 depicts the temporal trajectories of the propensity for social learning in populations where individuals maintain all expressed variants in their cultural repertoire.

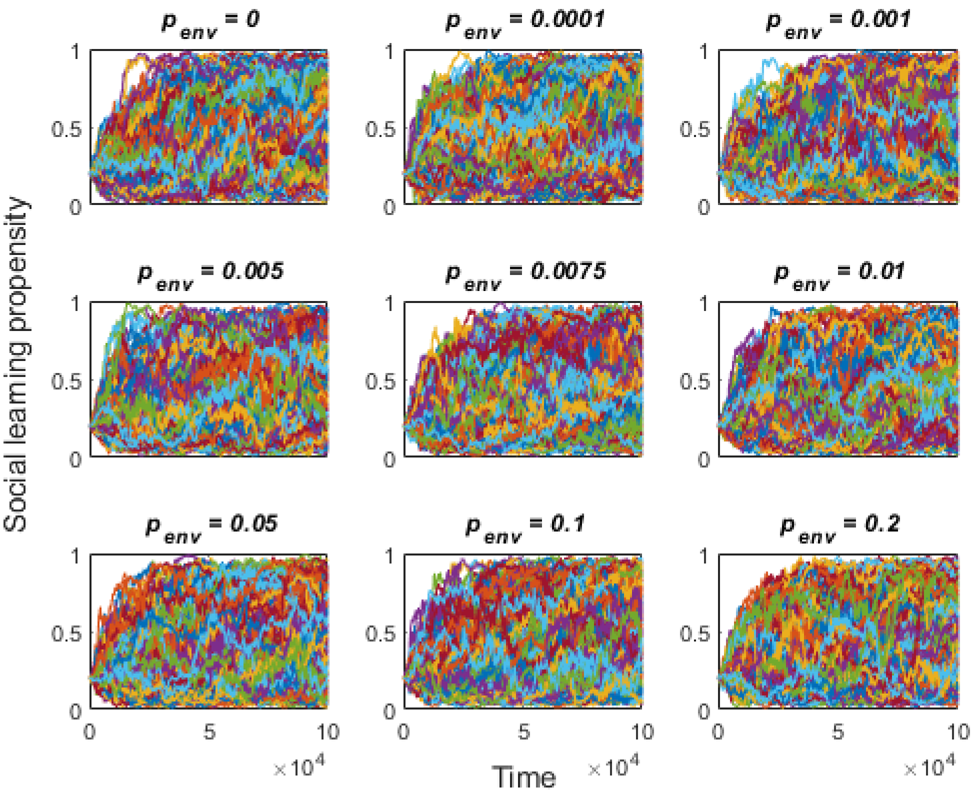

Figure S2: Temporal trajectories of social learning propensities. Each coloured line represents an independent trajectory of the evolution of the average propensity for social learning in scenario ii. For each p_{env}-value we ran 300 simulations.

For all simulations presented in the main text we assumed a population size of $N = 200$ individuals. To rule out any qualitative effect of this choice on the evolutionary outcomes, we compare in Fig. S3 the temporal trajectories of the propensities for social learning and forgetting and for the benefit levels of the population for $N = 200$ (pink lines) and $N = 1000$ (grey lines). To keep the number of generations constant at 500 in both cases, we increase the number of time steps to 500000 for $N = 1000$. We observe similar dynamics in populations of different size.

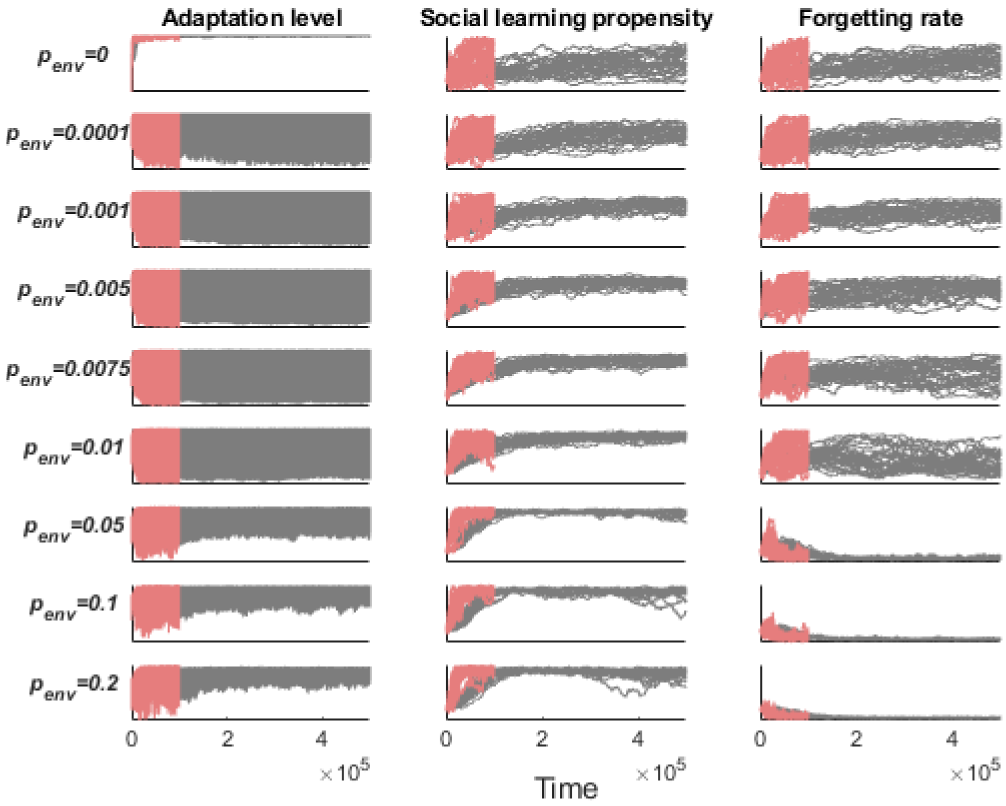

Figure S3: Temporal trajectories of the population's average benefit level (left column), average social learning propensity (middle column), and average forgetting propensity (right column) for $N = 200$ (pink lines) and $N = 1000$ (grey lines) based on 30 simulations.

S3 Alternative formulations of the forgetting process

Results in the main text where generated under the assumption that if forgetting occurs individual j chooses variant z to be removed from its repertoire with a probability, f_z^j, that is inversely proportional to the number of times j has expressed z so far, n_z^j. This probability is given by

$$f_z^j = \frac{A - n_z^j}{\sum\limits_{\substack{s=1 \\ s \neq i}}^{m^j} (A - n_s^j)} \tag{S1}$$

where $A = \sum\limits_{\substack{s=1 \\ s \neq i}}^{m^j} n_s^j$ and i denotes the variant expressed in this time step. To explore whether alternative formulations of the forgetting process change the results obtained in the main text we replaced Eq. (S1) with

- "Bad variant" forgetting: variants are forgotten inversely proprotional to their benefit level, a_s

$$f_z^j = \frac{a_z}{\sum\limits_{\substack{s=1 \\ s \neq i}}^{m^j} a_s},$$

- "Random" forgetting: all variants (apart from variant i expressed in this time step) are equally likely to be forgotten

$$f_z^j = \frac{1}{m^j - 1}.$$

Interestingly, neither of the above formulations changed the evolutionary trajectories for the propensities of social learning and for forgetting (see Fig. S4).

Further, repertoire structures (see Figs. S5,S6,S7), repertoire sizes and aspects of variant choice inaccuracy (see Fig. S8) reveal patterns similar to ones obtained under the original formulation of scenario iii.

Summarising, the number of expression events of a variant proves to be a good proxy for its level of benefit and consequently the forgetting process modelled by Eq. (S1) and "bad variant" forgetting result in very similar outcomes. Random forgetting, on the other hand, bears the danger of removing highly adaptive variants, independent of their numbers of previous expressions, from the repertoire. However, if this happens the individual will sooner or later engage in social learning which will add another highly beneficial variant to the repertoire. It is important that less beneficial variants can be removed not that the are preferentially removed.

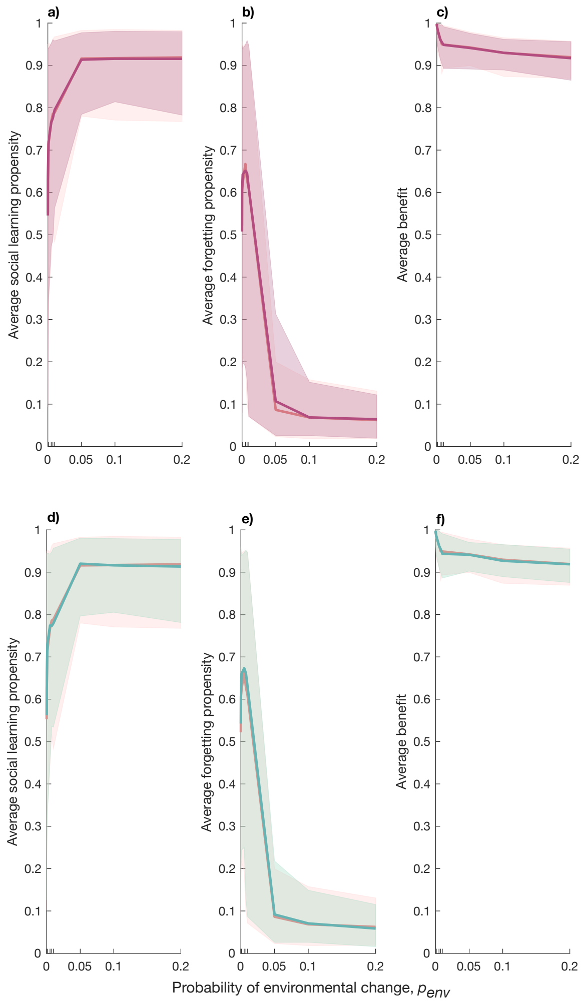

Figure S4: Relationship between rates of environmental change p_{env} and a,d) propensity for social learning, b,e) propensity for forgetting, and c,f) population-level benefit averaged over all individuals ($N = 200$) in the last time step for 300 independent simulations. Shaded areas represent 95% confidence intervals of population averages. Top row shows the comparison of results for scenario iii. (light pink) and scenario iii. with forgetting of bad variants (dark pink), and bottom row the comparison of results for scenario iii. (light pink) and scenario iii. with forgetting of random variants (green).

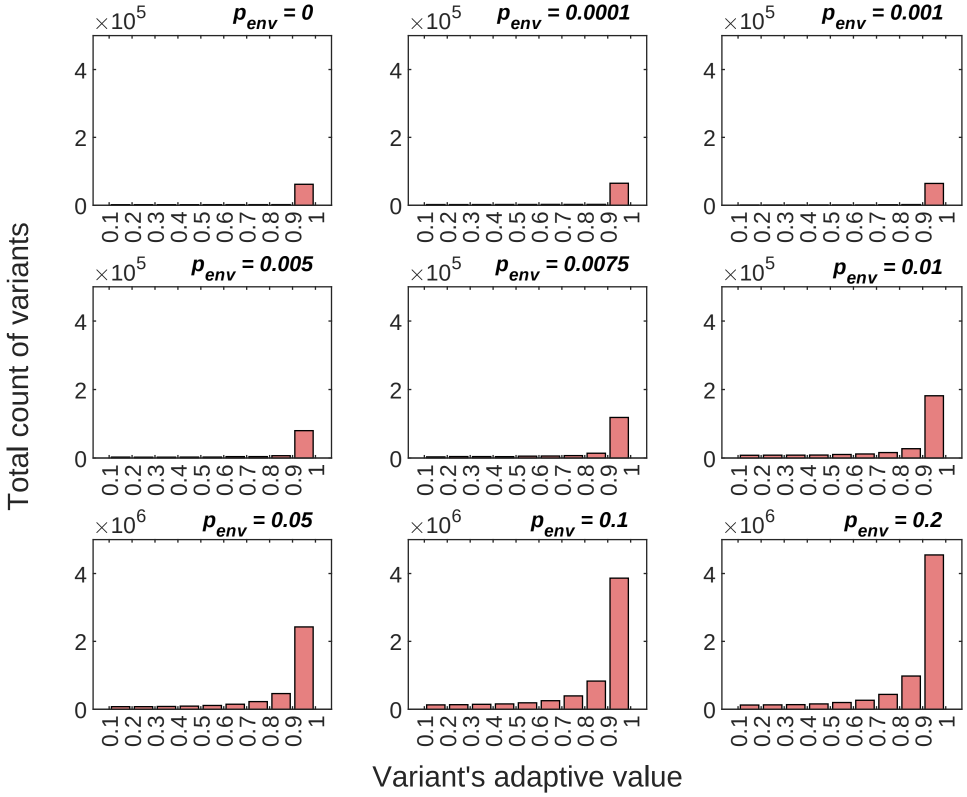

Figure S5: Histogram of the adaptation level of all variants contained in the repertoires of all individuals ($N = 200$) across 300 simulations for scenario iii. for different levels of environmental instability p_{env}. Note that the scale of the y-axes are enlarged for $p_{\text{env}} >= 0.05$.

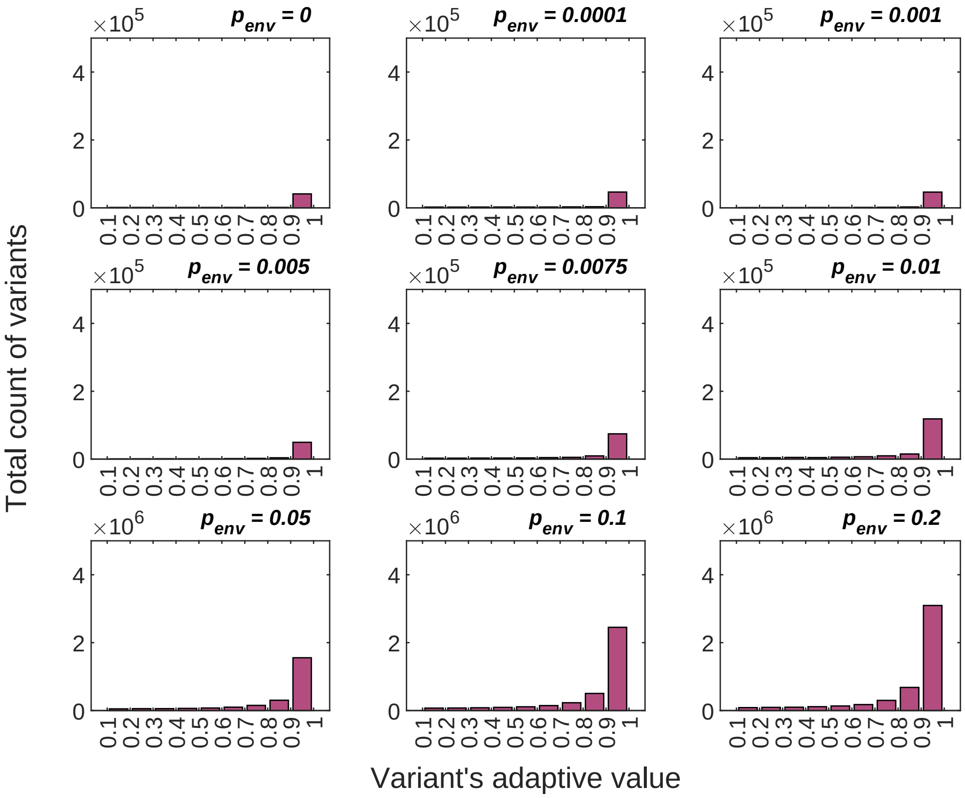

Figure S6: Histogram of the adaptation level of all variants contained in the repertoires of all individuals ($N = 200$) across 300 simulations for scenario iii. with "bad" variant forgetting for different levels of environmental instability p_{env}. Note that the scale of the y-axes are enlarged for $p_{\text{env}} >= 0.05$.

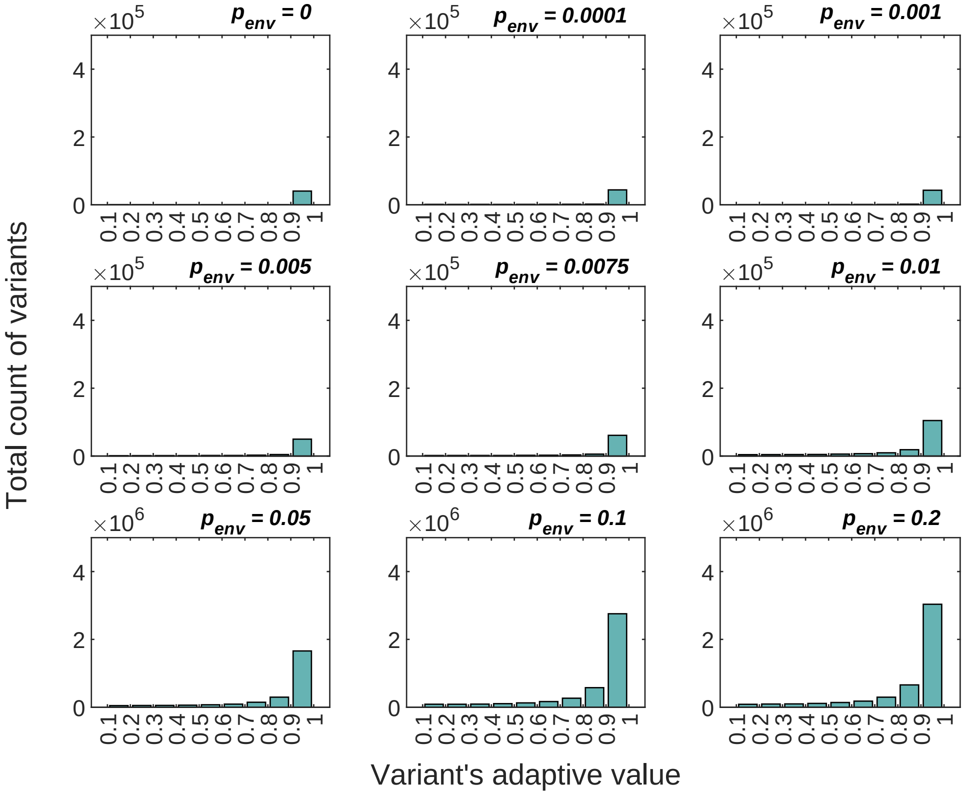

Figure S7: Histogram of the adaptation level of all variants contained in the repertoires of all individuals ($N = 200$) across 300 simulations for scenario iii. with random forgetting for different levels of environmental instability p_{env}. Note that the scale of the y-axes are enlarged for $p_{\text{env}} >= 0.05$.

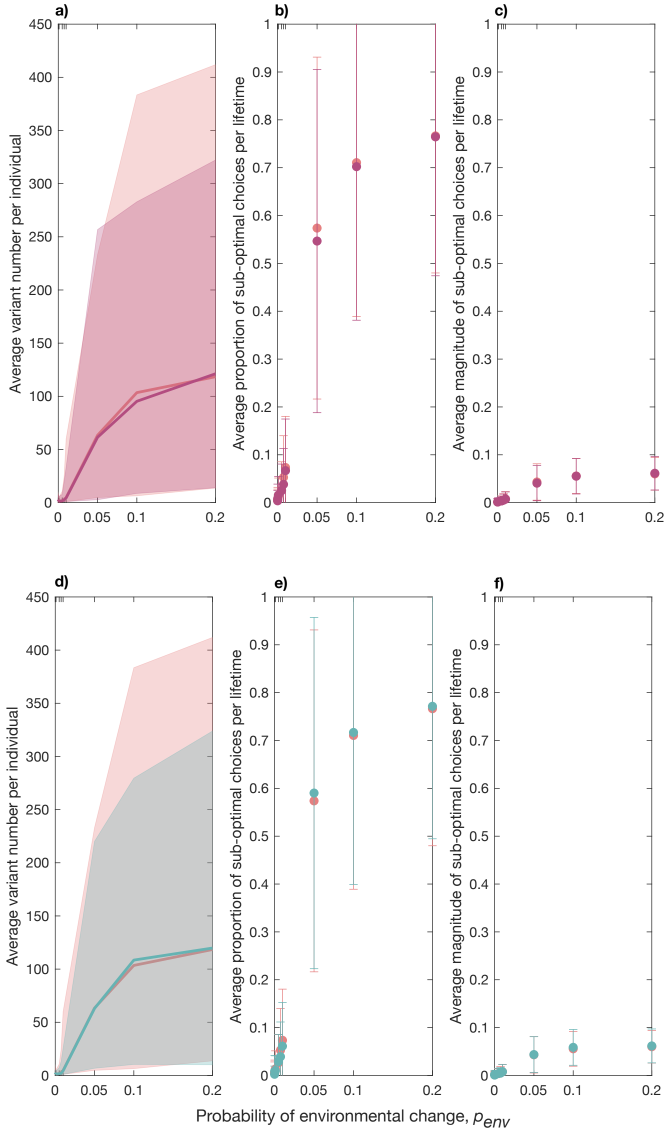

Figure S8: Relationship between rates of environmental change p_{env} and a,d) the size of individual repertoires, b,e) the proportion of sub-optimal choices, and c,f) the magnitude of sub-optimal choices relative to the most adaptive variant available in an individual's repertoire. Lines and dots represent the average across all individuals ($N = 200$) living in the last generation over 300 independent simulations. Shaded areas represent 95% confidence intervals of population averages. Errorbars indicate the standard deviation of population averages. Top row shows the comparison of results for scenario iii. (light pink) and scenario iii. with forgetting of bad variants (dark pink), and bottom row the comparison of results for scenario iii. (light pink) and scenario iii. with forgetting of random variants (green).

S4 Alternative formulation of the process of social learning

In the main text we assumed that social learning occurs in a payoff-biased way, i.e. individual j observes the levels of benefit expressed by all individuals during the last time step and chooses to copy a variant proportional to its observed benefit. In the following we want to explore whether the high propensities for social learning generated by scenario iii. (see Fig. 1a) are conditioned on this social learning process. To this end we replace payoff-biased social learning with unbiased social learning, i.e. individual j chooses at random one of the expressed cultural variants in the last time step.

We find that unbiased social learning can result in comparable evolutionary trajectories of the propensities for social learning and for forgetting, albeit slightly diminished population benefit levels (see Fig. S9).

Again we see that the individual cultural repertoires are highly structured (see Fig. S10) but contain more variants especially for higher p_{env}-values causing the frequency and magnitude of sub-optimal choices to increase (see Fig. S11). This in turn leads to more frequent expressions of less adaptive cultural variants. Once sub-optimal choices induce learning, social learners will more frequently copy sub-optimal variants since all individuals make frequent mistakes under this scenario. This reduces the efficiency of the unbiased social learning and eventually results in slightly reduced benefit levels of the population.

Summarizing, the fact that individual repertoires are highly structured not only through learning but due to the interplay of memory, variant choice and learning, i.e. that they contain substantially more highly adaptive cultural variants, makes the precise mechanism of the social learning process less relevant for the observed evolutionary outcome.

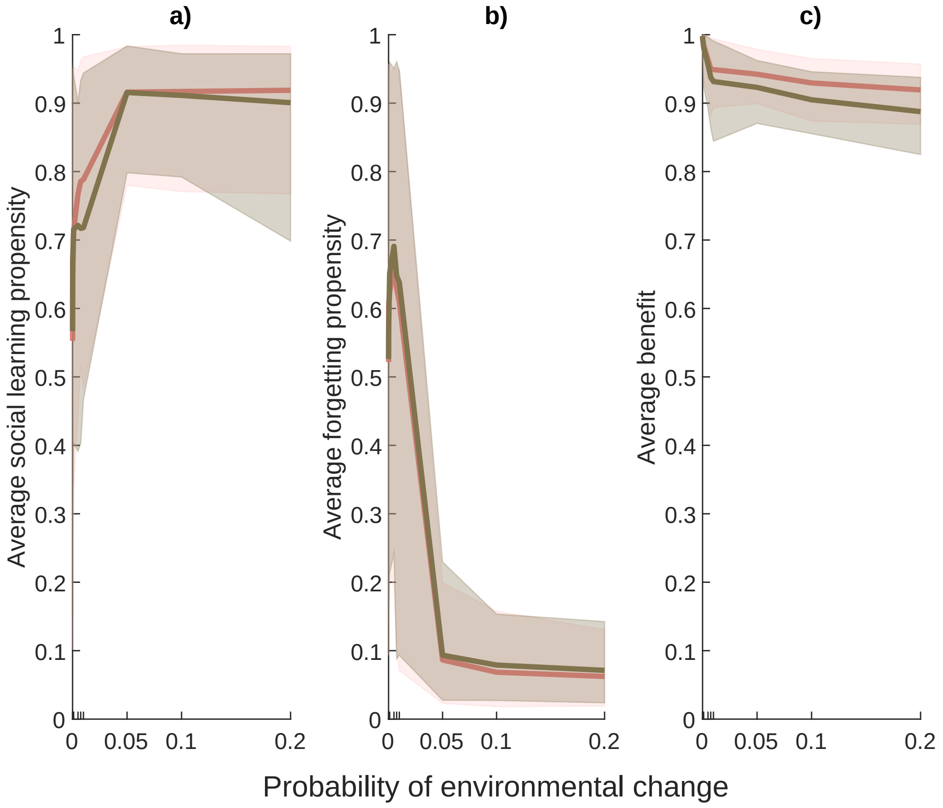

Figure S9: Relationship between rates of environmental change p_{env} and a) propensity for social learning, b) propensity for forgetting, and c) population-level benefit averaged over all individuals in the last time step for 300 independent simulations. Shaded areas represent 95% confidence intervals of population averages. Scenario iii. is shown by light pink lines and scenario iii. with unbiased social learning by olive lines.

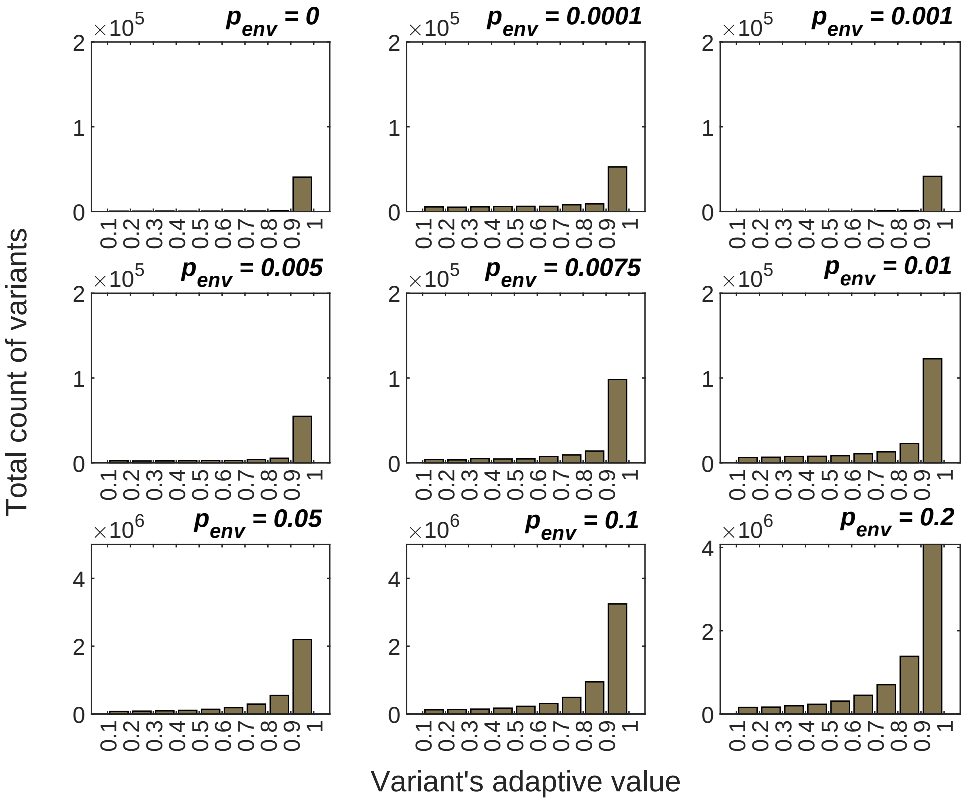

Figure S10: Histogram of the adaptation level of all variants contained in the repertoires of all individuals ($N = 200$) across 300 simulations for scenario iii. with unbiased social learning for different levels of environmental instability p_{env}. Note that the scale of the y-axes are enlarged for $p_{\text{env}} >= 0.05$.

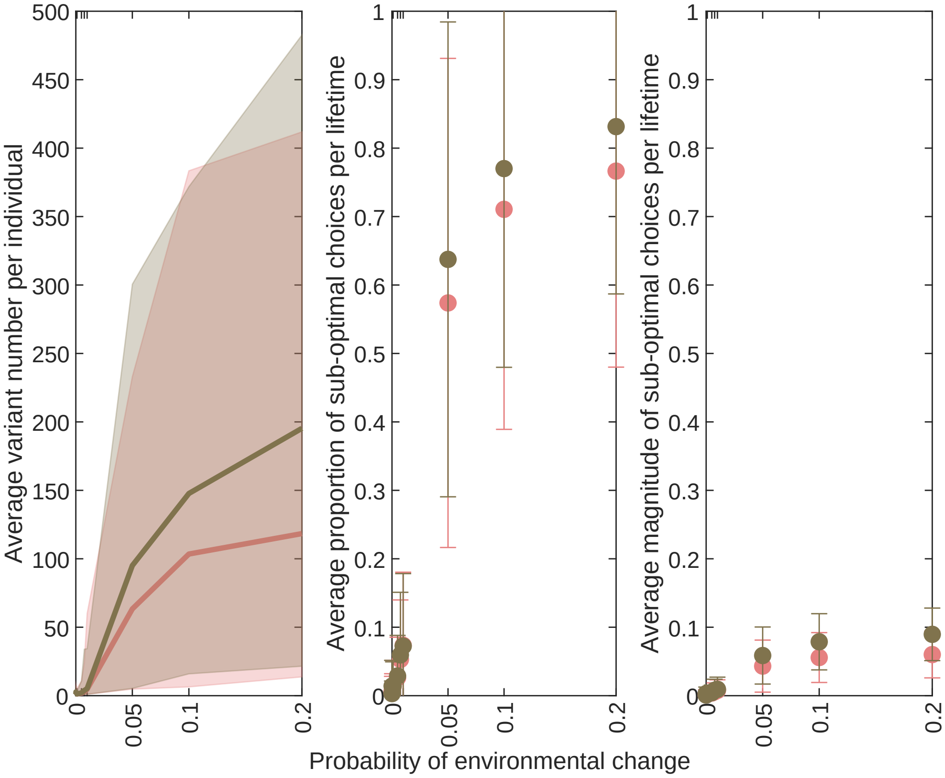

Figure S11: Relationship between rates of environmental change p_{env} and a) the size of individual repertoires, b) the proportion of sub-optimal choices, and c) the magnitude of sub-optimal choices relative to the most adaptive variant available in an individual's repertoire. Lines and dots represent the average across all individuals ($N = 200$) living in the last generation over 300 independent simulations. Shaded areas represent 95% confidence intervals of population averages. Errorbars indicate the standard deviation of population averages. Results are shown for scenario iii. (light pink) for scenario iii. with bad variant forgetting (dark pink), and scenario iii. with unbiased social learning (olive).

S5 Alternative formulation of how to choose a variant from the cultural repertoire

In the main text we assumed that individuals apply an error-prone decision rule when choosing a variant from their cultural repertoire. More precisely, individual j chooses variant $i \in M^j(t)$ with probability $p_i^j(k)$ which is proportional to variant i's level of benefit $a_i(k)$ relative to the rest of the repertoire in the following way

$$p_i^j(k) = \frac{a_i(k)}{\sum\limits_{s=1}^{m^j} a_s(k)}. \tag{S2}$$

If the cultural repertoire M^j of individual j is large decision rule (S2) will lead to frequent sub-optimal choices (as seen for scenario ii. in Fig. 4 in the main text). Of course, alternative decision heuristics, in particular more precise ones, are plausible, too. The field of reinforcement learning — studying, broadly speaking, learning what to do to maximize the received reward — has proposed a number of alternative decision rules.

In the following we explore the consequences when replacing rule (S2) with the softmax rule

$$p_i^j(k) = \frac{exp(a_i(k)/\tau)}{\sum\limits_{s \in M^j} exp(a_s(k)/\tau)}. \tag{S3}$$

The parameter τ controls the sensitivity towards differences in the benefit values: low values resulted in an almost deterministic choice of the variant with the highest benefit, whereas high values lead to choices almost independent from benefit levels. In the following we compare the cultural dynamics generated by scenario iii. with rule (S3) and $\tau = 0.1$ to the one generated by rule (S2).

Fig. S12 shows that a less error-prone way of choosing the 'best' variant from the cultural repertoire can have an impact on the evolutionary dynamic. First we see that the average benefit levels of the population are higher, especially for more unstable environments. The forgetting propensities evolve to lower values for all p_{env}-values leading to slightly larger individual repertoires (see Fig. S14a). Further, these repertoires are less structured: they contain a larger fraction of poorly adapted cultural variants (cf. Figs. S5 and S13). The social learning propensities are higher for low values of p_{env} but lower for high values of p_{env}. The increased social learning propensity in relatively stable environments is caused precisely by the higher repertoire sizes. For example, for $p_{\text{env}} = 0$ decision rule (S2) leads to a situation where almost all repertoires consist of a single, nearly perfectly adapted variant. However, under decision rule (S3) the repertoire size has increased to on average four variants. Even though (S3) is less error-prone than (S2) occasional sub-optimal choices do occur but they can be compensated by relying on social learning (see Fig. S15, compare green and light pink dots for low p_{env}). Consequently, social learning propensities evolve to high values. In contrast, for unstable environments repertoires grow large but nevertheless the magnitude of sub-optimal choices decreases in comparison to our main results (compare Fig. S14c), green and light pink dots) leading to fewer learning events during an individual's life time (see Fig. S15, compare green and light pink dots for high p_{env}).

The uncertainty in variant choice imposed by the parameter τ induces both sub-optimal choices and subsequent learning events somehow independent of repertoire size and structure. As a consequence of the reduction in learning events, social learning propensities evolve mainly through drift.

Decision rule (S3) chooses highly adaptive variants from the repertoire with a high probability. Consequently the size and structure of the repertoire has a much smaller effect on the efficiency of choice and therefore

forgetting can evolve to lower propensities which in turn shield from the 'accidental' forgetting of highly adaptive variants.

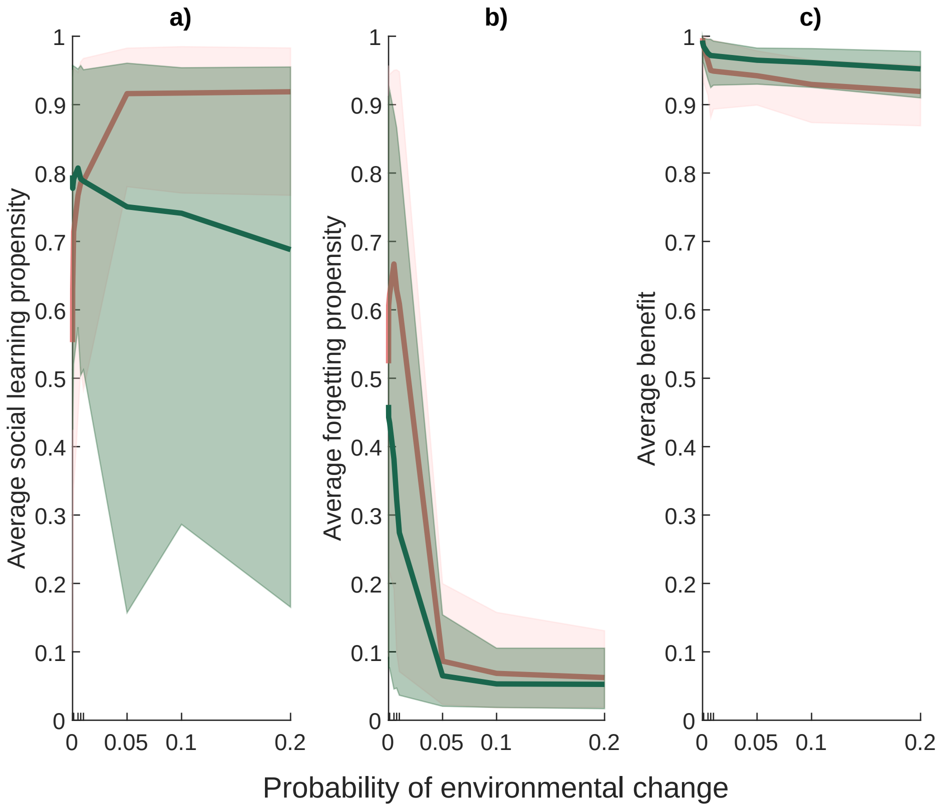

Figure S12: Relationship between rates of environmental change p_{env} and a) propensity for social learning, b) propensity for forgetting, and c) population-level benefit averaged over all individuals in the last time step for 300 independent simulations. Shaded areas represent 95% confidence intervals of population averages. Scenario iii. is shown by light pink lines and scenario iii. with variant choice according to the softmax rule (S3) by dark green lines.

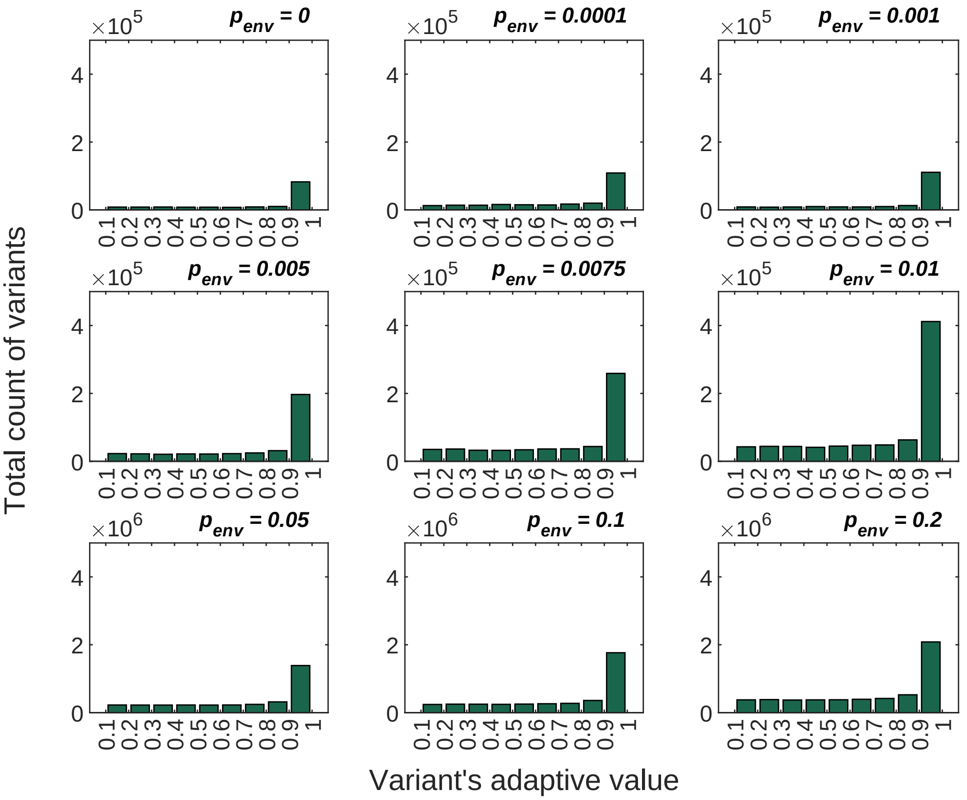

Figure S13: Histogram of the adaptation level of all variants contained in the repertoires of all individuals ($N = 200$) across 300 simulations for scenario iii. with variant choice according to the softmax rule (S3) for different levels of environmental instability p_{env}. Note that the scale of the y-axes are enlarged for $p_{\text{env}} >= 0.05$.

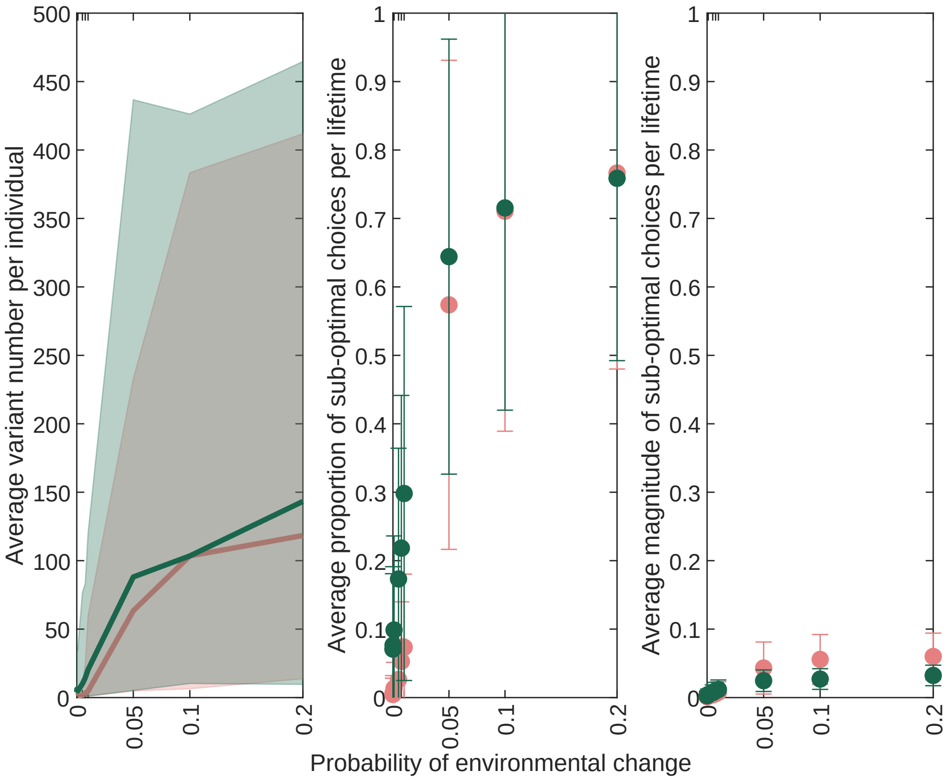

Figure S14: Relationship between rates of environmental change p_{env} and a) the size of individual repertoires, b) the proportion of sub-optimal choices, and c) the magnitude of sub-optimal choices relative to the most adaptive variant available in an individual's repertoire. Lines and dots represent the average across all individuals ($N = 200$) living in the last generation over 300 independent simulations. Shaded areas represent 95% confidence intervals of population averages. Errorbars indicate the standard deviation of population averages. Results are shown for scenario iii. (light pink) and scenario iii. with variant choice according to the softmax rule (S3) (dark green).

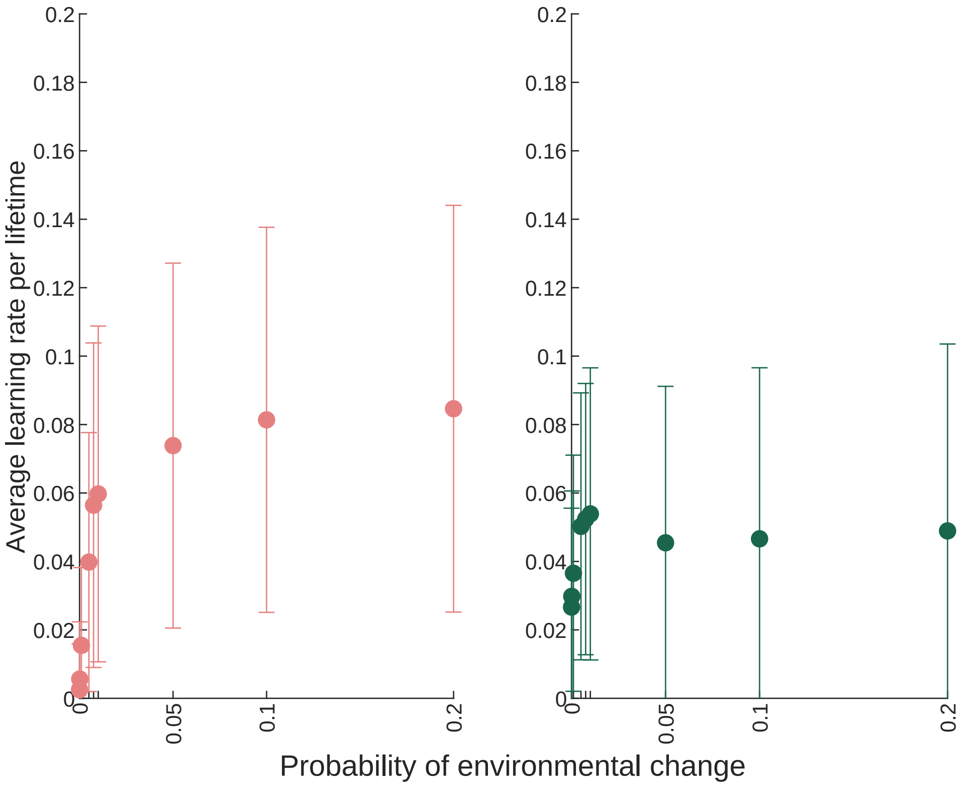

Figure S15: Relationship between rates of environmental change p_{env} and the rate of learning during an individual's life time. Dots show the average proportion of learning events during an individuals life time. Errorbars indicate the standard deviation of population averages. Results are shown for scenario iii. (light pink), and scenario iii. with variant choice according to the softmax rule (S3) (dark green).

S6 Alternative formulation of learning probabilities

In the main text we assumed that an individual engages in learning — as opposed to expressing the variant chosen from its repertoire — with a probability that depends on the expected benefit of the chosen variant, i: with probability $a_i(k)$ the individual expresses variant i and with probability $1 - a_i(k)$ it learns. In the following, we explore the consequences of replacing this assumption by a constant learning probability: independent of the expected benefit of the chosen variant individuals engage in learning with probability 0.2.

Fig. S16 shows that this fixed rate of learning has a strong effect on the evolutionary dynamic. First, we see that the average benefit levels of the populations are lower compared to scenario iii. for all p_{env}-values.

The fact that all individuals have to express the variant chosen from their repertoires through decision rule (S2) in, on average, 80% of the time steps leads to a weakening of the correlation between number of expressions and adaptation level of the variants. Consequently, the process of forgetting as operationalised by Eq. (S1) does not mainly remove poorly adapted cultural variants from the individual repertoires. This means it is more beneficial for individuals to forget less compared to scenario iii. (see Fig. S16). While this increases the proportion of sub-optimal choices as well as the magnitude of those choices (see Fig. S18) it substantially reduces the risk of forgetting highly adaptive variants.

The social learning propensities evolve to high values for relatively low p_{env}-values, i.e. for relatively stable environments, and decrease for higher values of p_{env}. We have seen in the main text that in stable environments scenario iii. leads to only a few learning events during an individual's life time. This, however, is not the case here as all individuals engage in learning (at least as long as their repertoire sizes do not reach the limit of 500) in, on average, 20% of the time steps. Due to the structuring of the individual repertoires (see Fig. S17) social learning is preferred over innovation. Higher p_{env}-values generate larger individual repertoire sizes (due to the low forgetting propensities) even to the point that a fraction of the population has reached the limit of 500 variants and therefore does not engage in learning anymore. This implies that — for this fraction of the population — there is no selection pressure acting on the social learning propensity which in turn slightly lowers the population-level propensity.

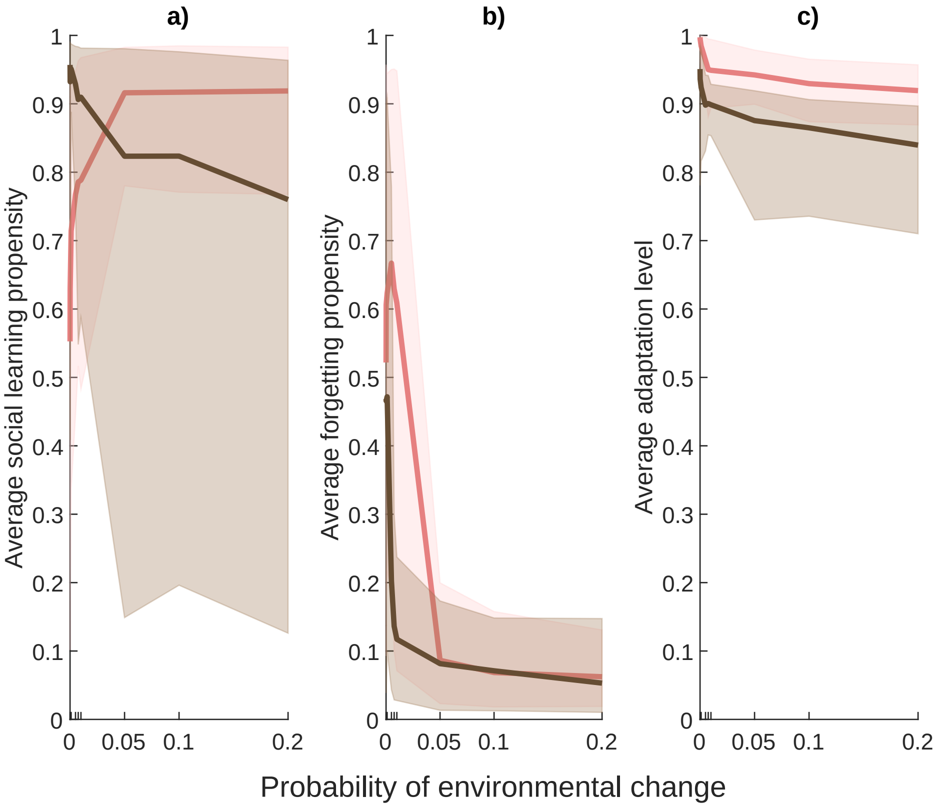

Figure S16: Relationship between rates of environmental change p_{env} and a) propensity for social learning, b) propensity for forgetting, and c) population-level benefit averaged over all individuals in the last time step for 300 independent simulations. Shaded areas represent 95% confidence intervals of population averages. Scenario iii. is shown by light pink lines and scenario iii. with constant learning probability of 0.2 by brown lines.

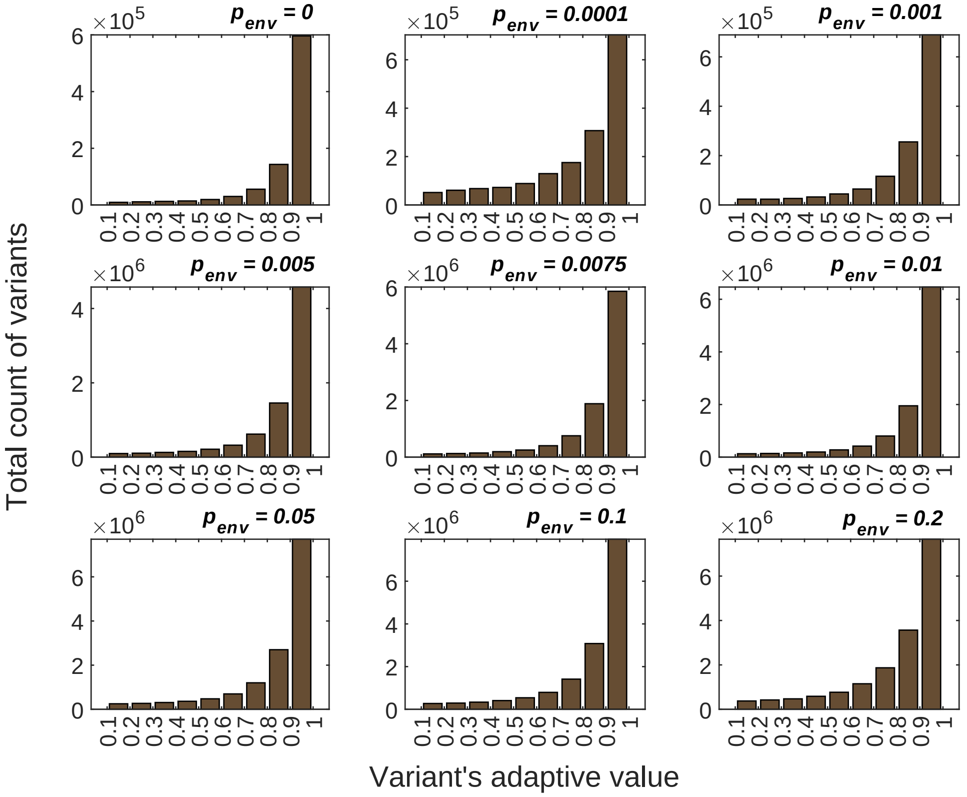

Figure S17: Histogram of the adaptation level of all variants contained in the repertoires of all individuals ($N = 200$) across 300 simulations for scenario iii. with constant learning probability of 0.2 for different levels of environmental instability p_{env}. Note that the scale of the y-axes are enlarged for $p_{\text{env}} >= 0.05$.

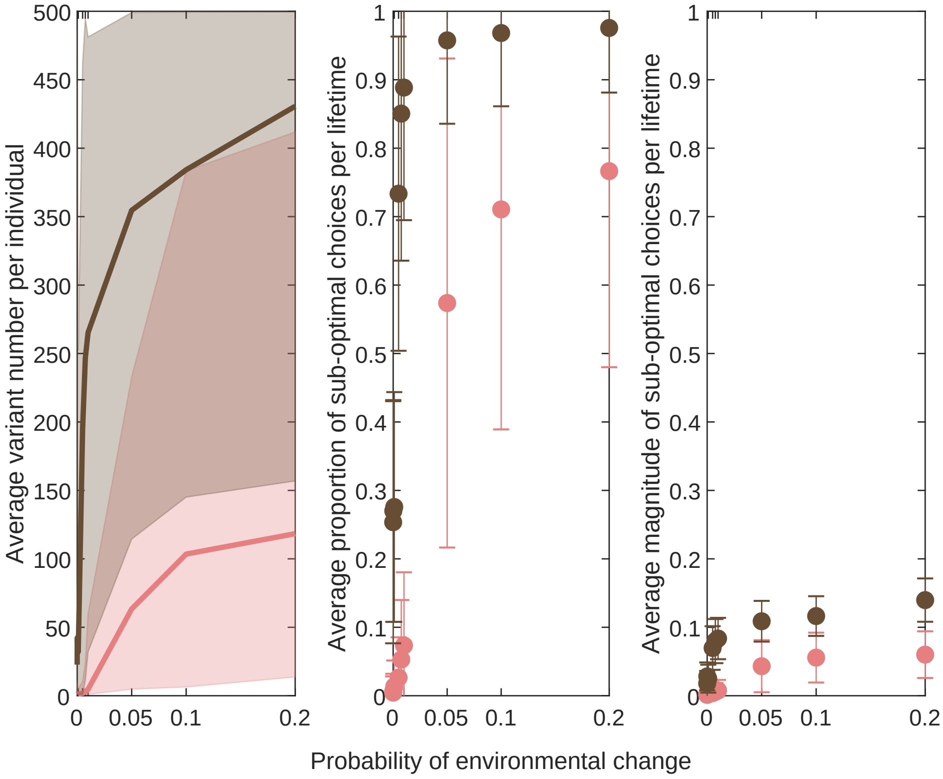

Figure S18: Relationship between rates of environmental change p_{env} and a) the size of individual repertoires, b) the proportion of sub-optimal choices, and c) the magnitude of sub-optimal choices relative to the most adaptive variant available in an individual's repertoire. Lines and dots represent the average across all individuals ($N = 200$) living in the last generation over 300 independent simulations. Shaded areas represent 95% confidence intervals of population averages. Errorbars indicate the standard deviation of population averages. Results are shown for scenario iii. (light pink) and scenario iii. with constant learning probability (brown).

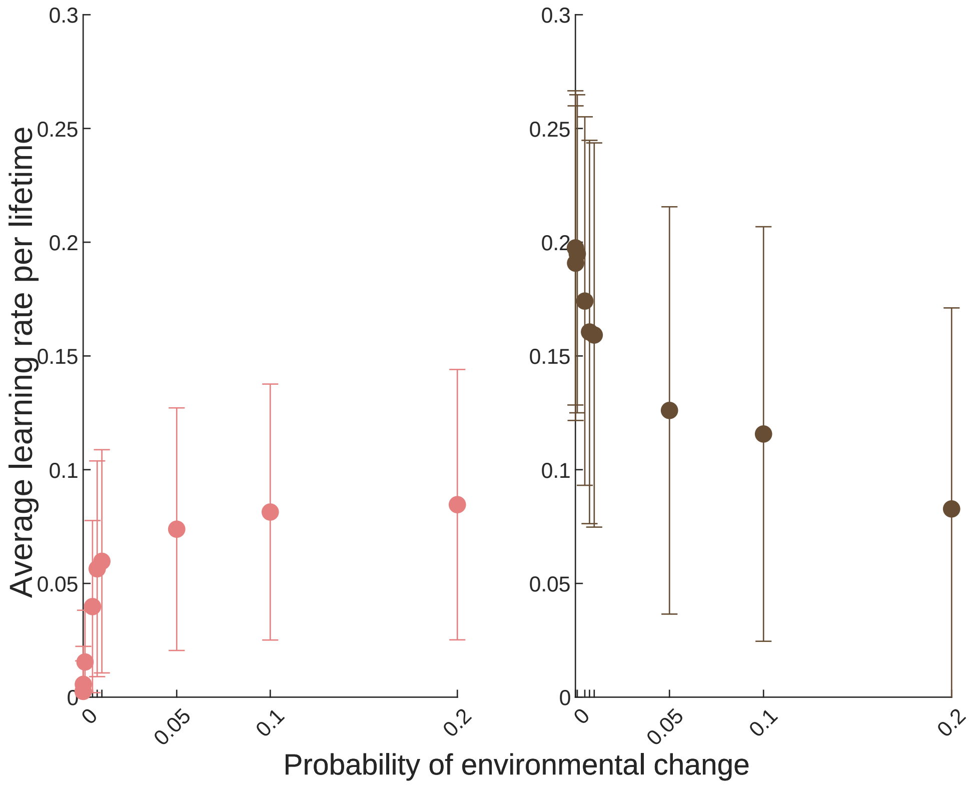

Figure S19: Relationship between rates of environmental change p_{env} and the rate of learning during an individual's life time. Dots show the average proportion of learning events during an individuals life time. Errorbars indicate the standard deviation of population averages. Results are shown for scenario iii. (light pink), and scenario iii. with constant learning probability of 0.2(brown).
